# Metabolic processes of *Methanococcus maripaludis* and potential applications

**DOI:** 10.1186/s12934-016-0500-0

**Published:** 2016-06-10

**Authors:** Nishu Goyal, Zhi Zhou, Iftekhar A. Karimi

**Affiliations:** Department of Chemical and Biomolecular Engineering, National University of Singapore, 4 Engineering Drive 4, Singapore, 117585 Singapore; School of Civil Engineering and Division of Environmental and Ecological Engineering, Purdue University, 550 Stadium Mall Drive, West Lafayette, IN 47907 USA

**Keywords:** *Methanococcus maripaludis*, Methanogen, Systems biology, Hydrogenotroph, Metabolism, Carbon capture and utilization, Nitrogen fixation

## Abstract

*Methanococcus maripaludis* is a rapidly growing, fully sequenced, genetically tractable model organism among hydrogenotrophic methanogens. It has the ability to convert CO_2_ and H_2_ into a useful cleaner energy fuel (CH_4_). In fact, this conversion enhances in the presence of free nitrogen as the sole nitrogen source due to prolonged cell growth. Given the global importance of GHG emissions and climate change, diazotrophy can be attractive for carbon capture and utilization applications from appropriately treated flue gases, where surplus hydrogen is available from renewable electricity sources. In addition, *M. maripaludis* can be engineered to produce other useful products such as terpenoids, hydrogen, methanol, etc. *M. maripaludis* with its unique abilities has the potential to be a workhorse like *Escherichia coli* and *S. cerevisiae* for fundamental and experimental biotechnology studies. More than 100 experimental studies have explored different specific aspects of the biochemistry and genetics of CO_2_ and N_2_ fixation by *M. maripaludis*. Its genome-scale metabolic model (*i*MM518) also exists to study genetic perturbations and complex biological interactions. However, a comprehensive review describing its cell structure, metabolic processes, and methanogenesis is still lacking in the literature. This review fills this crucial gap. Specifically, it integrates distributed information from the literature to provide a complete and detailed view for metabolic processes such as acetyl-CoA synthesis, pyruvate synthesis, glycolysis/gluconeogenesis, reductive tricarboxylic acid (RTCA) cycle, non-oxidative pentose phosphate pathway (NOPPP), nitrogen metabolism, amino acid metabolism, and nucleotide biosynthesis. It discusses energy production via methanogenesis and its relation to metabolism. Furthermore, it reviews taxonomy, cell structure, culture/storage conditions, molecular biology tools, genome-scale models, and potential industrial and environmental applications. Through the discussion, it develops new insights and hypotheses from experimental and modeling observations, and identifies opportunities for further research and applications.

**Electronic supplementary material:**

The online version of this article (doi:10.1186/s12934-016-0500-0) contains supplementary material, which is available to authorized users.

## Background

Methanococci are non-pathogenic, strictly anaerobic, hydrogenotrophic archaebacteria isolated from marine environments. Some are mesophilic, and others are thermophilic or hyperthermophilic [1]. The mesophilic methanococci are divided into four species: *Methanococcus maripaludis*, *M. vannielii*, *M. voltae*, and *M. aeolicus* [2]. In this article, we focus on *M. maripaludis*, whose type strain *M. maripaludis* JJ was isolated from salt marsh sediments in South Carolina [3]. Thenceforth, numerous strains have been isolated from estuarine sites in South Carolina, Georgia, and Florida [4]. Table 1 lists the characteristics of five fully sequenced strains (S2, C5, C6, C7 and X1).

**Table 1:** Characteristic features of *M. maripaludis* strains

Strains	Source/Habitat	Substrate	Optimum temperature	Optimum pH	Mol % GC	Growth rate (/h)	Sequenced by	Total genome size (Mbp)	ORFs
*M. maripaludis* S2	Salt marsh sediment [4]	Formate, or H_2_ and CO_2_	38 °C	6.8–7.2	34.4 ± 0.1	0.30	Hendrickson et al. [41]	1661	1772
*M. maripaludis* C5	Airport Marsh [4]	Formate, or H_2_ and CO_2_	35–40 °C	6–8	33.1 ± 0.1	0.21	Copeland et al. [121]	1789	1889
*M. maripaludis* C6	Roger’s Marsh [4]	Formate, or H_2_ and CO_2_	35–40 °C	6–8	34.2 ± 0.1	0.06	Copeland et al. [121]	1744	1888
*M. maripaludis* C7	Roger’s Marsh [4]	Formate, or H_2_ and CO_2_	35–40 °C	6–8	33.7 ± 0.1	0.20	Copeland et al. [121]	1772	1855
*M. maripaludis* X1	Thermophilic saline oil reservoir [122]	Formate, or H_2_ and CO_2_	NA	NA	32.9 ± 0.1	NA	Wang et al. [123]	1746	1892

*NA* not available

*M. maripaludis* is a fast growing mesophilic microbe with a doubling time of 2 h and optimum growth temperature at 38 °C. It reduces CO_2_ to methane via a modified Wood-Ljungdahl pathway, also known as Wolfe cycle [5]. Unlike other microorganisms that need complex carbon substrates such as pentoses, hexoses, alcohols, and their derivatives for their growth, *M. maripaludis* can use a simple substrate such as CO_2_ as the sole carbon source, and N_2_ as the sole nitrogen source [6]. However, it needs H_2_ or formate (HCOOH) for energy [1, 7, 8]. In other words, given a renewable source of H_2_, *M. maripaludis* has the potential to capture and convert the main source of global climate concerns, namely the CO_2_ emissions, into a useful fuel (methane).

Over the years, *M. maripaludis* S2 has become a well-studied model organism in the literature [9, 10] with well-developed genetic tools. However, in spite of more than 100 publications exploring its genetics and biochemistry, a comprehensive review of its metabolic processes is missing in the literature. This article presents a holistic and integrated view of its metabolic processes, and suggests some potential applications for this promising organism. In this article, we divide the entire metabolism of *M. maripaludis* into eight subsystems. We discuss six of them in detail in the main text, but defer, for the sake of brevity, the remaining two along with other relevant topics such as taxonomy and cultivation to Additional file 1. For each subsystem, we describe the key steps and their salient features, and identify the existing gaps in the metabolism. Then, we discuss molecular biology tools for manipulating the genome of *M. maripaludis*, and some limited systems biology work. Finally, we highlight potential applications for *M. maripaludis*.

## *Methanococcus maripaludis*—cell structure

**Fig. 1:**
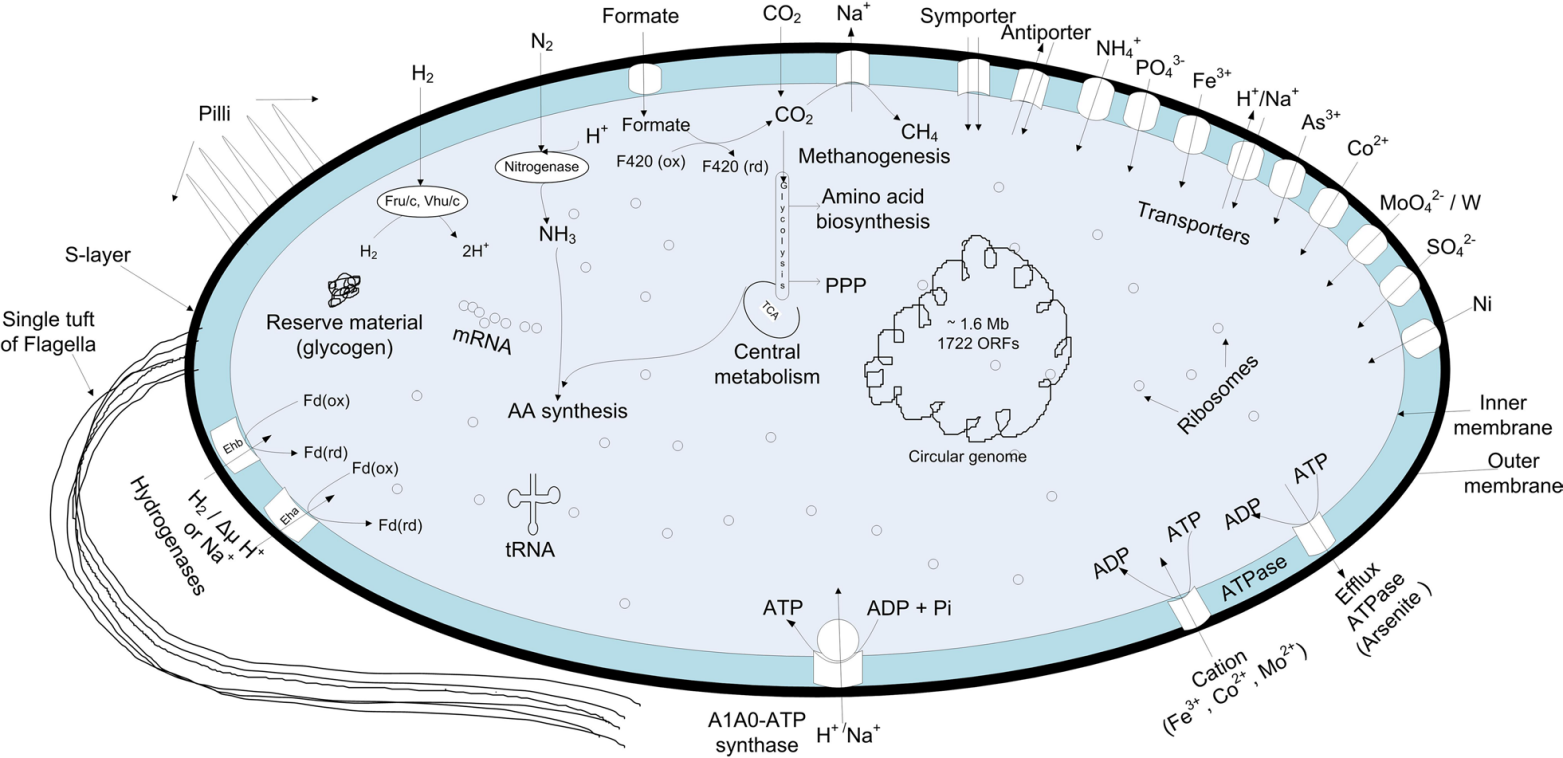
A schematic representation of a typical *M. maripaludis* cell

Figure 1 shows a simplistic view of the *M. maripaludis* cell. It is a weakly-motile coccus of 0.9–1.3 µm diameter [3]. This non-spore forming mesophile grows best between 20 and 45 °C at a pH ranging from 6.5 to 8.0 [11]. As in Fig. 1, its cell wall is a single, electron-dense, proteinaceous S-layer lacking peptidoglycan molecules. The S-layer proteins and flagellins have been discussed in the literature [12, 13]. Its cell wall lyses rapidly in low concentrations of detergents [3] and low-osmolarity buffers [14], which makes the isolation of DNA easier. Despite the differences in the presence of some amino acids, the primary structure of S-layer proteins shows a high degree of identity (38–45 %) with other microbes [15]. Ether lipids recovered from *M. maripaludis* mainly include glycolipids [14.2 mg ether lipid/g dry cell weight (DCW)] and polar lipids (0.4 mg ether lipid/g DCW) [16]. The motility in methanococci is due to the presence of flagella, however strong attachments by *M. maripaludis* to various surfaces require both flagella and pili [17]. Although the specific roles of the pili in *M. mariplaudis* are still unknown [18], if archaeal pili are similar to their bacterial counterparts, then they could be involved in functions related to cell-to-cell twitching, motility, attachment, biofilm formation, etc.

**Fig. 2:**
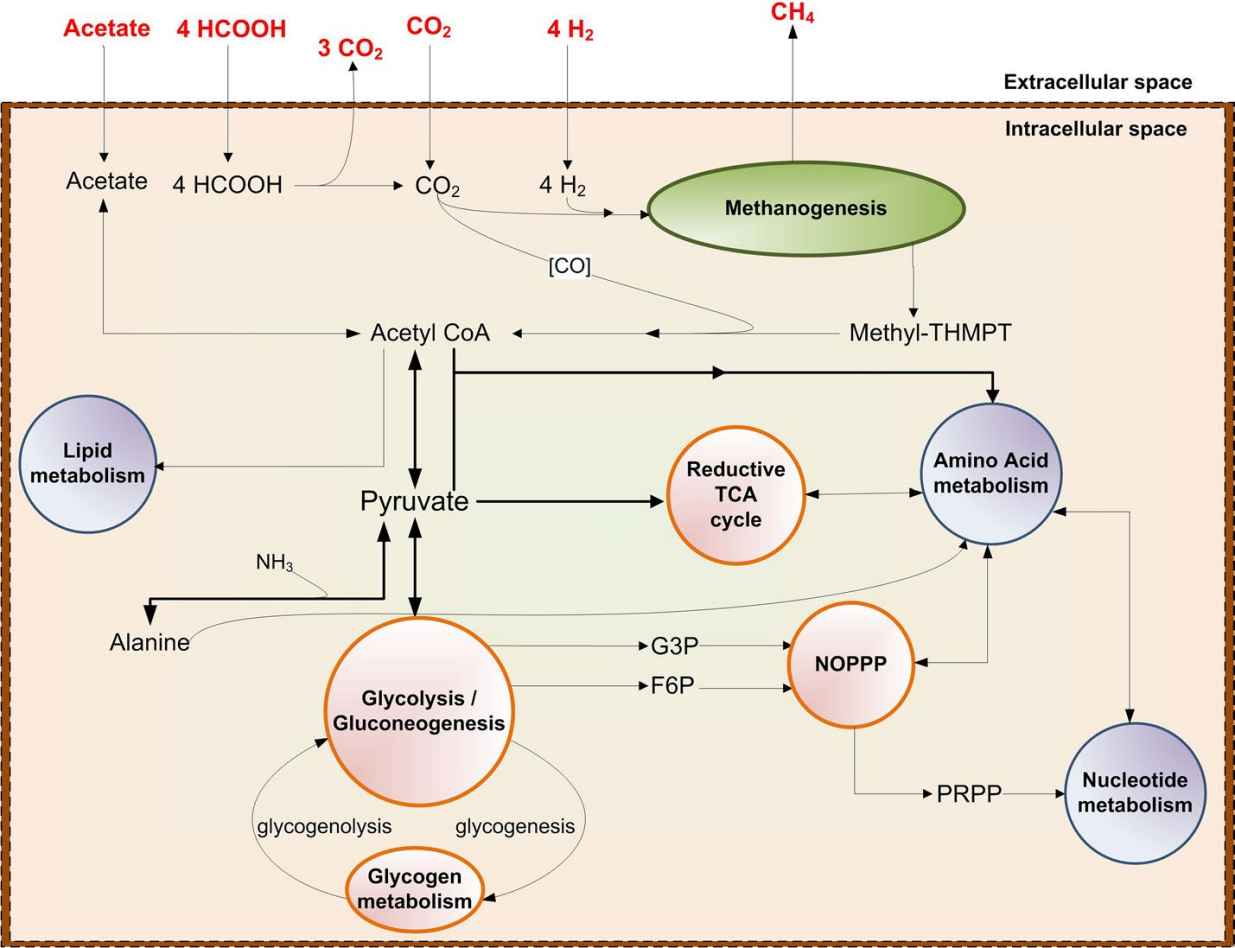
Simplistic overview of eight major metabolic subsystems in *M. maripaludis*. *HCOOH* formate, [*CO*] enzyme-bound CO, *Methyl-THMPT* methyl-tetrahydromethanopterin, *Acetyl-CoA* acetyl coenzyme A, *TCA* tricarboxylic acid cycle, *NOPPP* non-oxidative pentose phosphate pathway, *PRPP* 5-Phosphoribosyl diphosphate, *G3P* glyceraldehyde-3-phosphate, *F6P* fructose-6-phosphate

Figure 2 provides a comprehensive and consolidated picture of the metabolic system of *M. maripaludis*. As shown, it has eight major subsystems: methanogenesis, reductive tricarboxylic acid (RTCA) cycle, non-oxidative pentose phosphate pathway (NOPPP), glucose/glycogen metabolism, nitrogen metabolism, amino acid metabolism, and nucleotide metabolism. Methanogenesis, or the reduction of CO_2_ to methane, being its only pathway for energy generation, forms the foundation for its survival and growth [19, 20]. In other words, both methanogenesis and cell growth compete for the carbon source. The remaining seven subsystems provide the essential precursors for cell growth via two key intermediates: acetyl CoA and pyruvate. We now discuss the first six subsystems in detail.

## Methanogenesis

**Fig. 3:**
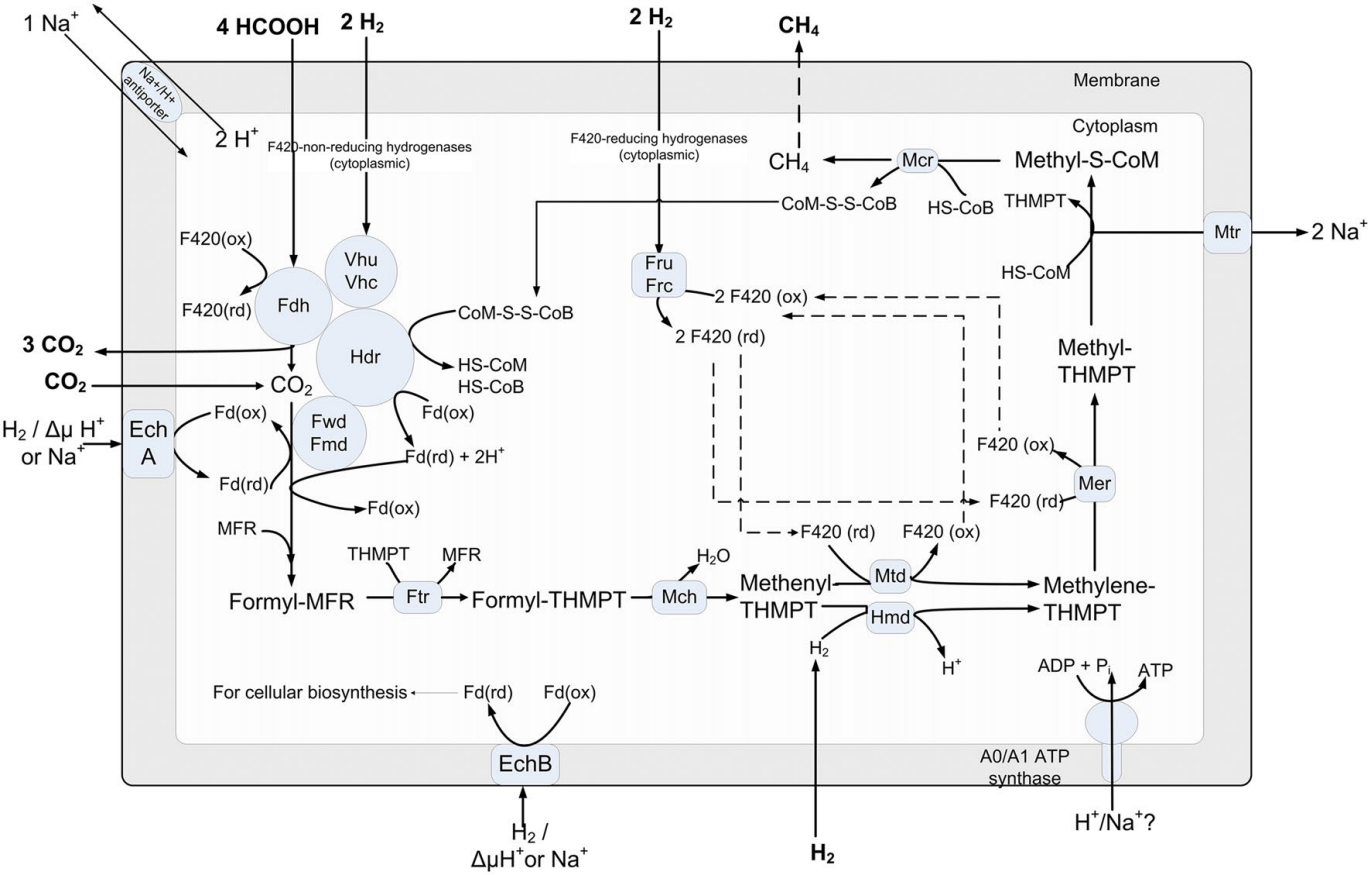
Energy producing pathway in *M. maripaludis.* *F420* coenzyme F420, *Vhu*/*Vhc* F420 non-reducing hydrogenase, *Fru*/*Frc* F420 reducing hydrogenase, *Fdh* formate dehydrogenase, *Hdr* heterodisulfide reductase, *Fwd/Fmd* tungsten/molybdenum containing formylmethanofuran dehydrogenase, *EchA* energy-converting hydrogenase A, *EchB* energy-converting hydrogenase B, *Fd(ox)* oxidized ferredoxin, *Fd (rd)* reduced ferredoxin, *Ftr* formyltransferase, *THMPT* tetrahydromethanopterin, *Mch* methyleneTHMPT cyclohydrolase, *Mtd* methyleneTHMPT dehydrogenase, *Hmd* 5,10-methenylTHMPT hydrogenase, *Mer* methyleneTHMPT reductase, *Mtr* methyltransferase, *Mcr* methyl-COM reductase, *HS-COM* coenzyme M (2-mercaptoethanesulfonate), *Methyl-S-COM* 2-(Methylthio)coenzymeM, *SH-CoB* thio-coenzyme B, *COM-S-S-COB* coenzyme M 7-mercaptoheptanoylthreonine-phosphate heterodisulfide

Methanogenesis (Fig. 3) is the biological production of methane via the reduction or disproportionation of relatively simpler carbon substrates such as CO_2_, formate, acetate, and methanol as follows.

$${\text{CO}}_{ 2} + {\text{ 4H}}_{ 2} \to {\text{ CH}}_{ 4} + {\text{ 2H}}_{ 2} {\text{O}}\quad \quad \Delta {\text{G}}^{0} = - 1 3 1 {\text{ kJ}}/{\text{mol}}$$

$$4 {\text{HCOOH }} \to {\text{ 3CO}}_{ 2} + {\text{ CH}}_{ 4} + {\text{ 2H}}_{ 2} {\text{O}}\quad \quad \Delta {\text{G}}^{0} = - 1 1 9 {\text{ kJ}}/{\text{mol}}$$

$${\text{CH}}_{ 3} {\text{COOH }} \to {\text{ CH}}_{ 4} + {\text{ CO}}_{ 2} \quad \quad \Delta {\text{G}}^{0} = - 3 6 {\text{ kJ}}/{\text{mol}}$$

$$4 {\text{CH}}_{ 3} {\text{OH }} \to {\text{ 3CH}}_{ 4} + {\text{ CO}}_{ 2} + {\text{ 2H}}_{ 2} {\text{O}}\quad \quad \Delta {\text{G}}^{0} = - 10 6 {\text{ kJ}}/{\text{mol}}$$

While formate, acetate, and methanol can oxidize/reduce by themselves, CO_2_ needs an electron donor such as H_2_ [3], formate [21], or electricity [22]. In *M. maripaludis*, methanogenesis occurs via the reduction of CO_2_ with H_2_/formate/electricity, or the disproportionation of formate. Lohner et al. [22] demonstrated H_2_-independent electromethanogenesis from CO_2_ in both wild-type *M. maripaludis* strain S2 and hydrogenase mutant strain MM1284. The mutant strain under the same conditions showed a factor of 10 lower methane production rates as compared to the wild-type strain S2 [22]. However, their attempts to prove biomass growth were inconclusive.

The formate-dependent methanogenesis involves an additional endergonic step where formate is oxidized to CO_2_ via formate dehydrogenase with a simultaneous reduction of coenzyme F420 [23]. The reduced coenzyme F420 serves as the electron carrier for two intermediary steps in methanogenesis, but H_2_ is probably not an intermediate [21, 24]. As shown in Fig. 3, the resulting CO_2_ feeds into the first step of methanogenesis.

A recent study [8] showed the effects of H_2_ and formate limitation/excess on growth yield and regulation of methanogenesis using a continuous culture of *M. maripaludis*. They concluded that the growth yield (g DCW/mol CH_4_) decreased remarkably with excess H_2_ or formate. While they speculated energy spilling or dissipation to be a possible cause, the exact cause is still unclear.

While *M. maripaludis* can also assimilate other carbon substrates, such as acetate and pyruvate, they are not physiologically relevant for methane production [25, 26]. No methane production from acetate (17), and extremely low methane from pyruvate [26] (only 1–4 % compared to that for H_2_) have been reported.

### Mechanism

The structures and functions of the cofactors and coenzymes involved in methanogenesis are listed in Table 2. The first step in methanogenesis is the reduction of CO_2_. It involves the simultaneous oxidation of low-potential reduced ferredoxins and capture of CO_2_ by methanofuran (MFR) to form formyl-MFR (ΔG^0^ = 0 kJ/mol) [27]. These extremely low- potential ferredoxins could come from two pools [28]. One is EchA that not only uses one H_2_, but also consumes proton-motive force (PMF) to generate ferredoxins. This accounts for only 4 % of the reduced ferredoxins as shown in a ∆5H_2_ase mutant [24]. The second and the major pool is Vhu/Hdr bifurcation complex that consumes two H_2_ and generates one pair of relatively high potential electrons to reduce CoB-S-S-CoM and another pair of extremely low potential electrons to reduce the ferredoxins. The formyl group from formyl-MFR is then transferred to THMPT (ΔG^0^ = −5 kJ/mol) to form formyl-THMPT, and the latter is then dehydrated to methenyl-THMPT (ΔG^0^ = −5 kJ/mol) [29]. In the next two steps, the reduced F420 gets oxidized by supplying electrons to reduce methenyl-THMPT to methylene-THMPT (ΔG^0^ = +6 kJ/mol) and methylene-THMPT to methyl-THMPT (ΔG^0^ = −6 kJ/mol) [30]. These reactions are fully reversible, as evidenced by their near-zero free energy changes. The oxidized F420 is then reduced (ΔG^0^ = −11 kJ/mol) in the presence of H_2_ [27]. Next, the methyl group from methyl-THMPT is transferred to coenzyme M (HS-CoM) in an exergonic step (ΔG^0^ = −30 kJ/mol) coupled with 2Na^+^ translocation by a membrane-bound enzyme complex [31]. This reaction builds up an electrochemical Na^+^ gradient, which drives energy production via ATP synthase [27]. The final step of methanogenesis is the reductive demethylation of methyl-S-CoM to methane and CoM-S-S-CoB (ΔG^0^ = −30 kJ/mol). Subsequently, this CoM-S-S-CoB gets reduced with the help of H_2_ to form HS-CoM and HS-CoB (ΔG^0^ = −39 kJ/mol) [32]. This reduction of CoM-S-S-CoB mediates via an electron bifurcation mechanism [27]. This step along with the earlier step involving the Na^+^ translocation supplies the major energy demand of *M. maripaludis*.

**Table 2:** Structure and functions of unusual coenzymes involved in methanogenesis of *M. maripaludis*

Cofactor/coenzyme	Structure	Function	Reference
Methanofuran		Carbon-carrier cofactors involved in the first step of the methanogenic reduction of CO_2_ to formyl-methanofuran	[124, 125]
Tetrahydro-methanopterin		Carries single carbon fragments between formyl and methyl oxidation levels in methanogens, typically formyl, methenyl, methylene or methyl group	[5, 125]
Coenzyme B		Function as electron carrier in metabolism of methanogens	[125, 126]
Coenzyme M		Required for methyl-transfer reactions in the metabolism of methanogens., HS-CoB reacts with Methyl-S-CoM to release methane and CoM-S-S-CoB in the final step of methanogenesis	[125, 127]
Coenzyme F420		Major electron transfer currency. Transfer electrons from H_2_ to the consecutive intermediates of methane biosynthesis e.g. coenzyme F420 hydrogenase, 5,10-methylene-THMPT reductase and methylene-THMPT dehydrogenase	[34, 125]
Cofactor F430		This cofactor is the prosthetic group of the methyl-CoM reductase which catalyzes the release of methane and CoB-S–S-CoM in the final step of methanogenesis	[125, 128]

### Hydrogenases

The key to the survival of *M. maripaludis* on CO_2_ is its ability to take up external H_2_ and generate electrons from ${\text{H}}_{ 2} \, \to {\text{ 2H}}^{ + } + {\text{ 2e}}^{ - }$ with the help of seven hydrogenases (Fig. 3). These are Fru, Frc, Vhu, Vhc, Hmd, EchA, and EchB, which can be categorized in different manners. The first five are cytoplasmic and the last two are membrane-bound [33]. Fru and Frc use cofactor F420 [34]; Vhu and Vhc use ferredoxin and CoM/CoB [35]; Hmd uses direct H_2_ [34]; and EchA and EchB use ferredoxins as electron carriers [24]. *M. maripaludis* needs four pairs of electrons to reduce one mole of CO_2_ to methane. Fru/Frc can supply two pairs, Vhu/Vhc can supply two pairs, Hmd can supply one pair, and EchA/EchB can supply one pair each.

Of the above, Fru/Frc and Vhu/Vhc play a major role in H_2_ uptake. Fru and Frc reduce two molecules of coenzyme F420 with the help of two H_2_ [34]. One F420 (rd) gets oxidized by reducing methenyl-THMPT and the other by reducing methylene-THMPT. Vhu and Vhc facilitate the flow of electrons from H_2_ to heterodisulfide reductase (Hdr) complex [35], which in turn catalyzes the reductions of CoM/CoB and ferredoxins via an electron bifurcation mechanism [32]. These reduced ferredoxins are the major electron suppliers during the first step of methanogenesis.

Hmd uses H_2_ to reduce methenyl-THMPT to methylene-THMPT [34] without any carrier. As shown in Fig. 3, Mtd also catalyzes the same reaction, but with the help of reduced F420 as an electron carrier. Hendrickson et al. [34] demonstrated that during the growth on H_2_ and CO_2_, Hmd is not essential in the presence of active Fru/Frc, but is essential otherwise. In contrast, the ΔFru, ΔFrc, and ΔHmd mutants grew normally during formate-dependent growth, proving that formate acted as the electron donor.

EchA generates a small portion of low-potential reduced ferredoxins required for the first step of methanogenesis. Its role in *M. maripaludis* is anaplerotic, because it is required only under certain conditions such as (1) to replenish the intermediates of methanogenesis cycle, and (2) imperfect coupling during electron bifurcation [24]. Lie et al. [24] showed this by eliminating all nonessential pathways of H_2_ metabolism and using formate as the sole electron donor. In this case, both Hdr complex and EchA independently provided the electrons for growth.

In contrast, EchB supplies electrons to anabolic oxidoreductases for the synthesis of precursors such as pyruvate and acetyl CoA [33, 36]. EchB mutants affect the autotrophic growth severely, but it is unclear how they still survive. When conditions limit growth, anabolic CO_2_ fixation is unimportant, but methanogenesis continues. Under such a scenario, EchA is essential, but EchB could be detrimental [24].

During formate-dependent growth, the H_2_ required for the essential anaplerotic (EchA) and anabolic (EchB) functions is produced from formate. This H_2_ production can occur via two pathways as demonstrated by Lupa et al. [23] in *M. maripaludis*. One involves Fdh1-Fru/Frc, and the other involves Fdh1-Mtd-Hmd. Of these two, the former seems to be predominant (~90 %), as the deletion of either Fdh1 or Fru/Frc reduced H_2_ production rates severely [23].

### Energy generation/conservation

In most organisms, electron movement along the cell membrane is the key to energy transduction. Substrate oxidation releases electrons that move along the membrane-bound cytochrome carriers and extrudes protons out of the cell to generate a potential gradient. The potential difference drives the protons back into the cell, while at the same time synthesizing ATP from ADP and Pi via ATP synthase [37]. Hydrogenotrophic methanogens such as *M. maripaludis* lack such an electron transport chain [38]. In place of cytochrome carriers, *M. maripaludis* uses methyl-THMPT: HS-COM methyltransferase (Mtr), the only membrane-bound enzyme complex in the core methanogenic pathway, to extrude Na^+^/H^+^ out of the cell [27, 31]. This creates a Na^+^/H^+^ ion motive force (positive outside), which on their translocations into the cell generate ATP via an A1A0-type ATP synthase [39]. However, a direct experimental evidence specifically for Na^+^ or H^+^ gradient does not exist in the literature. To conserve ATP, *M. maripaludis* uses reduced ferredoxins as low-potential electron carriers for the highly endergonic reduction of CO_2_ to formyl-MFR. As discussed earlier, these ferredoxins are supplied predominantly by the Hdr complex [32] and supplemented by EchA.

The genome sequence of *M. maripaludis* indicates the presence of membrane-bound A1A0-type ATPases (chimeric ATP synthases) instead of the F1F0-type ATPases found in Bacteria and Eukarya [40, 41]. The catalytic unit of the A1A0-type ATPase is structurally homologous to the V-type ATPase and functionally homologous to the F1F0-type ATPase [42]. But, the membrane-embedded motors in the A1A0-type ATP synthases are exceptional due to their novel functions and structural features [43].

## Acetyl-CoA synthesis

*M. maripaludis* can synthesize acetyl-CoA from either CO_2_ or acetate [7, 25]. The CO_2_-based synthesis occurs with the help of carbon monoxide decarbonylase/acetyl-CoA synthase complex (CODH/ACS) [7]. Sequencing studies have confirmed the existence of CODH/ACS in a single cluster (MMP0980-MMP0985) [41]. During the CO_2_-based synthesis, methyl-THMPT, an intermediate of methanogenesis, contributes the methyl carbon of acetyl-CoA, while the CO generated from the reduction of CO_2_ in the presence of reduced ferredoxins by CODH, contributes the carboxyl carbon [7]. The acetate-based synthesis is accomplished by AMP-forming acetate CoA ligase (MMP0148, *acs*A) in *M. maripaludis* [25]. Shieh et al. [25] showed that *M. maripaludis* can assimilate up to 60 % of its cellular carbon from exogenous acetate. A sequencing study [41] also showed the presence of ADP-forming acetyl-CoA synthetase gene (MMP0253, *acd*) in *M. maripaludis*, which catalyzes acetate formation and ATP synthesis from acetyl CoA, ADP and Pi. However, no literature study has experimentally demonstrated the biosynthesis of free acetate by *M. maripaludis*.

## Pyruvate synthesis

Pyruvate is the entry point into glycolysis, citric acid cycle, and amino acid metabolism. Acetyl-CoA is converted to pyruvate through pyruvate:ferredoxin oxidoreductases (PORs) [34, 44, 45] as follows:

$${\text{Acetyl-CoA }} + {\text{ CO}}_{ 2} + {\text{ 2 Fd}}\left( {\text{rd}} \right) \, + {\text{ 2H}}^{ + } \leftrightarrow {\text{ Pyruvate }} + {\text{ CoA }} + {\text{ 2 Fd}}\left( {\text{ox}} \right)$$

This is reversible in that PORs also catalyze pyruvate oxidation to acetyl-CoA in the absence of H_2_ [26]. However, pyruvate oxidizes very slowly in *M. maripaludis* and PORs appear to function mainly in the anabolic directions during growth [44].

The PORs containing five polypeptides in *M. maripaludis* are encoded by one gene cluster (*por*ABCDEF). Of these, *por*EF is unique to *M. maripaludis*, because the N-terminal sequences of the first four polypeptides (*por*ABCD) are similar to those in other *Archaea* [45]. The importance of *por*EF in *M. maripaludis* was highlighted by Lin et al. [46]. They showed that *por*EF mutants of *M. maripaludis* JJ grew extremely slowly and pyruvate-dependent methanogenesis was completely inhibited. Interestingly, *por*F mutant failed to restore growth, but restored methanogenesis to wild-type levels. In contrast, *por*E mutant restored growth partially, but did not restore methanogenesis. This indicates that *por*F serves as an electron donor to PORs.

Pyruvate is also a precursor for alanine biosynthesis via alanine dehydrogenase (MMP1513, *ald*) [47]. The same enzyme catalyzes the reverse reaction also, i.e. alanine to ammonia and pyruvate, in *M. maripaludis*. In addition, alanine transaminase that catalyzes the conversion of alanine to pyruvate in several organisms including *Pyrococcus furiosus*, *Escherichia coli*, *Mus musculus,* and *Homo sapiens*, may also exist in *M. maripaludis*. Our inference is based on a BLASTp search with the protein sequences of *M. maripaludis*. Our search located proteins with high similarity (e-value = 7e−63) to the alanine transaminase from *P. furiosus*. *M. maripaludis* uptakes alanine with the help of alanine permease and alanine racemase. While the former transports both l-alanine and d-alanine into the cell, the latter is essential for converting d-alanine to l-alanine [47–49], because alanine dehydrogenase is specific for l-alanine only.

## Glycolysis/gluconeogenesis and glycogenolysis/glyconeogenesis

*M. maripaludis* does not assimilate carbohydrates such as pentoses and hexoses, as it lacks the required transporters [41]. However, it has all the enzymes and cofactors required for glycolysis/gluconeogenesis and glycogenolysis/glyconeogenesis with some unique features. In fact, studies have shown that methanococci such as *M. maripaludis* synthesize and store glycogen as a reserve metabolite, and use it for methane generation in the absence of exogenous substrates [50]. The bifunctional activity of ADP-dependent phosphofructokinase (PFK)/glucokinase (GK) has been demonstrated experimentally in both *M. jannaschii* [51] and *M. maripaludis* [52]. Castro-Fernandez et al. [52] measured the activities of glucose phosphorylation versus dephosphorylation. They unexpectedly observed that the latter was two-folds more efficient than the former. Based on these observations, they indicated that *M. maripaludis* can catalyze d-glucose formation, and suggested a possibility of methane production from glycogen or d-glucose during starvation in *M. maripaludis*.

**Fig. 4:**
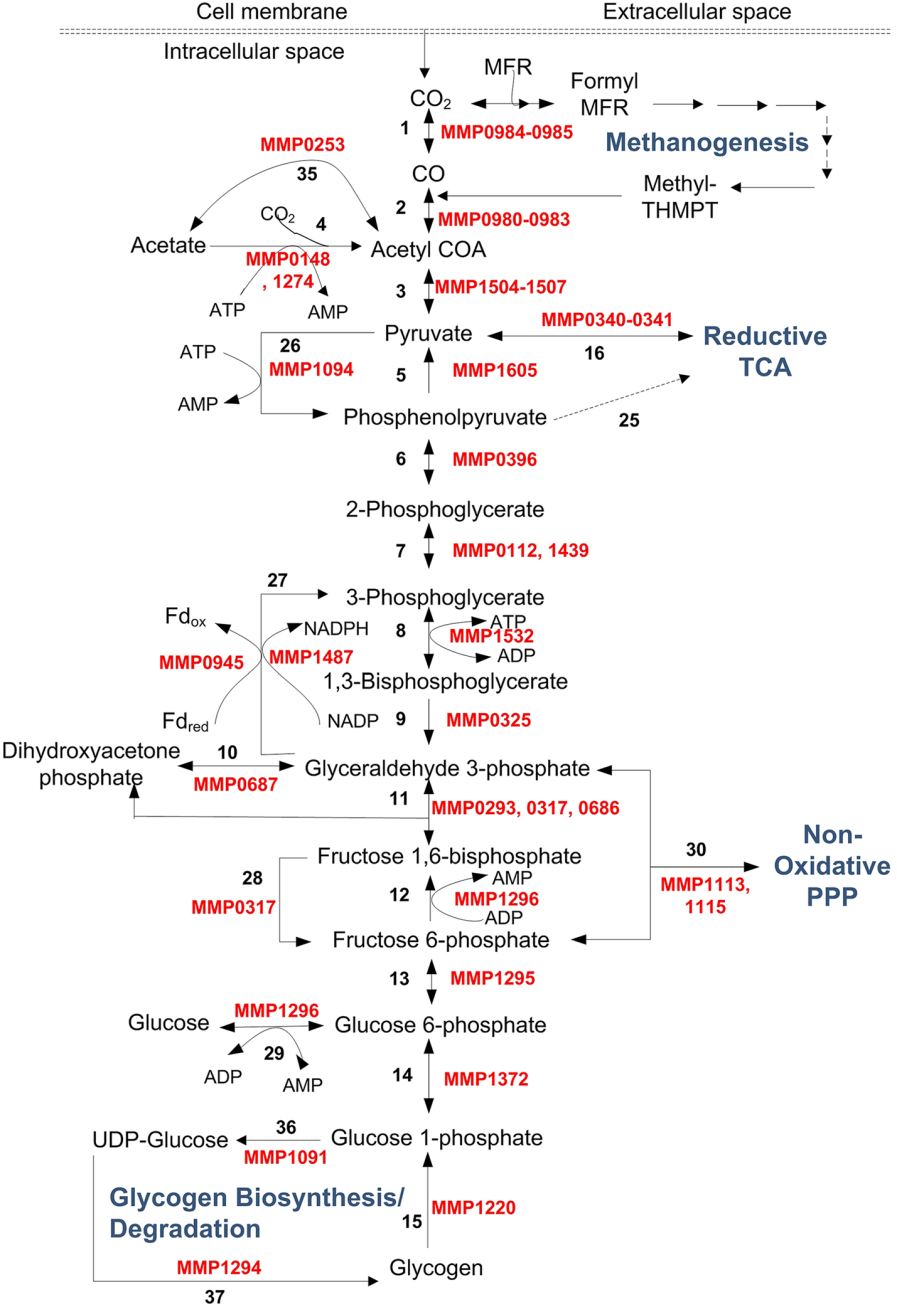
Glycolysis/gluconeogenesis and glycogenolysis/glyconeogenesis in *M. maripaludis* with associated ORFs. The *solid lines* show the presence of enzymes in *M. maripaludis* and dotted lines show their absence. The enzymes corresponding to the various reaction numbers are: *1*. carbon-monoxide dehydrogenase (1.2.7.4, *codh* & *por*EF); *2*. acetyl CoA decarbonylase (2.1.1.245, *acds*); *3*. pyruvate:ferredoxin oxidoreductase/synthase (1.2.7.1, *por*ABCD); *4*. acetyl CoA synthetase (AMP forming) (6.2.1.1, *acs*A); *5*. phosphenolpyruvate kinase (2.7.1.40, *pyk*); *6*. enolase (4.2.1.11, *eno*); *7*. 2,3-bisphosphoglycerate mutase (5.4.2.12, *pgm*); *8*. phosphoglycerate kinase (2.7.2.3, *pgk*); *9*. glyceraldehyde-3-phosphate dehydrogenase (1.2.1.59, *gapdh*); *10*. triosephosphate isomerase (5.3.1.1, *tpi*); *11*. fructose-bisphosphate aldolase (4.1.2.13. *fbp*); *12*. phosphofructokinase (2.7.1.147, *pfk*); *13*. glucose-6-phosphate isomerase (5.3.1.9, *pgi*); *14*. Phosphoglucomutase (5.4.2.8, *pgm*); *15*. glycogen phosphorylase (2.4.1.1, *glg*P); *16*. pyruvate carboxylase (6.4.1.1, *pyc*B); *25*. phosphoenolpyruvate carboxylase; *26*. phosphoenolpyruvate synthase (2.7.9.2, *pps*A); *27*. NADP-dependent glyceraldehyde-3-phosphate dehydrogenase (1.2.1.9, *gapn*) OR ferredoxin-dependent glyceraldehyde-3-phosphate dehydrogenase (1.2.7.6, *gapor*); *28*. fructose-bisphosphatase (3.1.3.11, *fbp*); *29*. ADP-specific phosphofructokinase (2.7.1.147, *pfk*); *30*. Transketoloase (2.2.1.1, *tkl*); *35*. Acetate CoA synthetase (ADP-forming) (6.2.1.13, *acd*); *36*. UTP-glucose-1-phosphate uridylyltransferase (2.7.7.9); *37*. starch synthase (2.4.1.21, *glg*A)

Unlike non-methanogenic *Archaea* that use ED pathway, *M. maripaludis* [50] uses a modified Embden-Meyerhof-Parnas (EMP) pathway with some unique features. These features include the reduction of ferredoxins instead of NAD (e.g. PORs and GAPOR) [53], ADP-dependent kinases [51], zero or very low ATP yields [54], highly divergent phosphoglucose isomerase [55], and phosphoglycerate mutase [56]. Of the eight enzymatic steps (5-13) from pyruvate to glucose-6-phosphate (Fig. 4), five are reversible and catalyzed by the same enzyme, while the rest are irreversible (5, 9, and 12). However, even for the three irreversible steps, reverse steps are catalyzed by alternative enzymes [phosphenolpyruvate synthase (PPS), glyceraldehyde-3-phosphate dehydrogenase (GAPDH), and fructose-bisphosphatase (FBP)]. In other words, all the steps leading to glucose-6-phosphate from pyruvate are reversible in principle. Glycogen in *M. maripaludis* is then synthesized by converting 1) glucose-6-phosphate to glucose-1-phosphate via phosphoglucomutase (MMP1372), 2) glucose-1-phosphate to UDP-glucose via UTP-glucose-1-phosphate uridylyltransferase (MMP1091), and 3) the growing polymeric chain of UDP-glucose to glycogen via glycogen synthase (MMP1294) with the release of UDP molecule [41]. On the other hand, glycogen in *M. maripaludis* degrades to glucose-6-phosphate by converting (1) glycogen to glucose-1-phosphate via glycogen phosphorylase (MMP1220), and (2) glucose-1-phosphate to glucose-6-phosphate via glucose phosphomutase (MMP1372) [41, 50]. The low activity of PFK in comparison to FBP in *M. maripaludis* suggests that glyconeogenesis is the predominant function of EMP pathway in *M. maripaludis* pointing to the storage of glycogen as a reserve material [50]. This predominance of the anabolic direction is further confirmed by the high activities of the reversible hexose phosphate conversions (via glucose phosphomutase, glucose-6-phosphate isomerase, and fructose-bisphosphate aldolase) and triose phosphate conversions for pentose biosynthesis (via enolase, 2, 3-bisphosphoglycerate mutase, and glyceraldehyde-3-phosphate dehydrogenase). Yu et al. [50] further showed that glycogen content increased from 0.11 % ± 0.05 % DCW (A_660_ ≤ 0.5) to 0.34 ± 0.19 % DCW (A_660_1.0–1.6) during growth, while glycogen consumption depended on the substrate for methanogenesis.

Given the key roles of glycolysis/gluconeogenesis in *M. maripaludis*, it is critical to understand their regulation. In general, a pathway can be regulated by (1) substrate availability, (2) up- or down-regulating enzyme activities for rate-limiting steps, (3) allosteric regulation of enzymes, and (4) covalent modifications such as phosphorylations of substrates. Essentially, the enzymes catalyzing the irreversible steps are most suited for regulation [57]. In most *Archaea*, nonphosphorylating NADP^+^-dependent glyceraldehyde-3-phosphate (G3P) dehydrogenase (GAPN), phosphorylating glyceraldehyde-3-phosphate dehydrogenase (GAPDH), and glyceraldehyde-3-phosphate ferredoxin oxidoreductase (GAPOR) act as the regulatory points in glycolysis [58–60]. The genome sequence of *M. maripaludis* codes for all three genes, namely GAPN (MMP1487), GAPDH (MMP0325), and GAPOR (MMP0945) [41]. GAPOR catalyzes ferredoxin-dependent G3P oxidation, GAPN catalyzes NADP-dependent G3P oxidation, and GAPDH catalyzes G3P synthesis. Based on the activity, transcriptomic, and flux balance analyses in *M. maripaludis*, Park et al. [61] showed that GAPOR is a post-transcriptionally regulated enzyme that is completely inhibited by the presence of 1 µM ATP, and (unlike GAPN) is most likely involved only under non-optimal growth conditions.

Yu et al. [50] mentioned pH-dependent PFK (optimum pH = 6.0) as an important regulatory enzyme in *M. maripaludis*. The activation and inhibition of PFK was found to be dependent on the presence/absence of various substrates such as ADP, AMP, Pi, cAMP, and citrate. Yu et al. [50] also reported that full activity of pyruvate kinase, another key enzyme in glycolysis, depended on Mn^2+^. In contrast to Mn^2+^, Fe^2+^ showed 70 % activity, and Mg^2+^ showed 20 % activity of pyruvate kinase, while Zn^2+^, Cu^2+^, Co^2+^, and Ni^2+^ showed zero activity. The activity of phosphoglycerate mutase was unaffected by Mg^2+^ and AMP, and depended on the presence of reduced dithiothreitol, cysteine hydrochloride, and glutathione.

## Tri-carboxylic acid (TCA) cycle

**Fig. 5:**
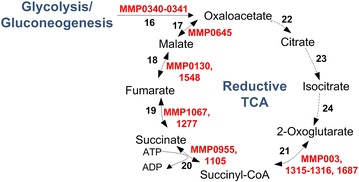
Reductive tricarboxylic acid cycle in *M. maripaludis* with associated ORFs. The *solid lines* show the presence of enzymes in *M. maripaludis* and *dotted lines* show their absence. The enzymes corresponding to the various reaction numbers are: *16*. pyruvate carboxylase (6.4.1.1, *pyc*B); *17*. malate dehydrogenase (1.1.1.37, *mdh*) *18*. fumarate hydratase (4.2.1.2, *fum*A); *19*. succinate dehydrogenase/fumarate reductase (1.3.5.4/1.3.4.1, *sdh*A); *20*. succinyl-CoA synthetase (6.2.1.5, *suc*C & *suc*D); *21*. 2-oxoglutarate oxidoreductase (1.2.7.3, *kor*ABDG); *22*. citrate synthase; *23*. aconitate; *24*. isocitrate dehydrogenase

TCA cycle plays an important role in generating electron carriers such as NADH & FAD for energy production [62]. Most aerobes have an oxidative TCA cycle to oxidize complex carbon molecules, such as sugars, to CO_2_ and H_2_O to generate energy [62]. However, most anaerobes have RTCA cycles to reduce CO_2_ and H_2_O to synthesize carbon compounds. Methanogens being anaerobes also have RTCA cycles. Furthermore, their TCA cycles are incomplete, as they lack several steps and enzymes [63]. *M. maripaludis* in particular lacks phosphoenolpyruvate carboxykinase, citrate synthase, aconitate, and isocitrate dehydrogenase [25, 64]. The missing steps in *M. maripaludis* are shown as dashed lines in Fig. 4. As shown in Fig. 4, pyruvate is the entry metabolite in *M. maripaludis* for TCA cycle. In the absence of phosphenolpyruvate carboxylase (PPC), *M. maripaludis* converts pyruvate to oxaloacetate via pyruvate carboxylase (PYC). Oxaloacetate is then reduced to 2-oxoglutarate via a series of intermediates (Malate, Fumarate, Succinate, Succinyl CoA) in the TCA cycle as shown in Fig. 5. Hendrickson et al. [41] reported the complete genome sequence of *M. maripaludis* and noted that 2-oxoglutarate oxidoreductase, the last enzyme in the TCA cycle, has four subunits (MMP0003, MMP1315, MMP1316, and MMP1687) that are not contiguous. This is in contrast to PORs that are also oxidoreductases, but have contiguous subunits (MMP1502-MMP1507).

Regulation of TCA cycle in *M. maripaludis* in particular, and *Archaea* in general, is poorly understood. However, 2-oxoglutarate plays an important role in nitrogen regulation [65]. In *M. maripaludis*, NrpR protein represses nitrogen fixation in ammonia-rich conditions by binding to the *nif* promoters [66]. In the absence of ammonia, 2-oxoglutarate is unable to synthesize glutamate, hence its level increases. High levels of 2-oxoglutarate act as the inducer and prevent binding of NrpR to *nif* promoters, resulting in the activation of nitrogen fixation and glutamine synthetase to bring down 2-oxoglutarate levels.

The TCA regulation in *Methanobacterium thermoautotrophicum,* another methanogen with an incomplete reductive cycle, can shed some light on the regulation in *M. maripaludis*. As reported by Eyzaguirre et al. [30] for *M. thermoautotrophicum,M. maripaludis* may also exhibit unidirectional synthesis of phosphenolpyruvate via phosphenolpyruvate synthetase (*pps*A). The activity of this enzyme may be inhibited by AMP, ADP, and 2-oxoglutarate. Similarly, PYC, the ATP-dependent enzyme responsible for pyruvate carboxylation in *M. maripaludis*, may exhibit anabolic function as reported by Mukhopadhyay et al. [30] in *M. thermoautotrophicum.* Furthermore, its activity may depend on biotin, ATP, Mg^2+^ (or Mn^2+^, Co^2+^), pyruvate, and bicarbonates; and it may be inhibited by ADP and 2-oxoglutarate.

## Pentose phosphate pathway (PPP)

**Fig. 6:**
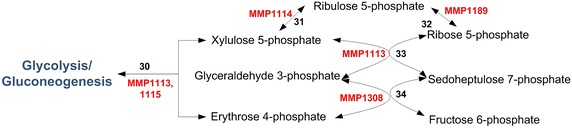
Non-oxidative pentose phosphate pathway in *M. maripaludis* with associated ORFs. The enzymes corresponding to the various reaction numbers are: *30*. Transketoloase (2.2.1.1, *tkl*); *31*. ribulose-phosphate 3-epimerase (5.1.3.1, *rpe*); *32*. ribose-5-phosphate isomerase (5.3.1.6); *33*. transketoloase (2.2.1.1, *tkt*); *34*. translaldolase (2.2.1.2, *tal*)

PPP is essential for the syntheses of nucleotides and nucleic acids in *M. maripaludis*. Glyceraldehyde-3-phosphate and fructose-6-phosphate synthesized during glycolysis/gluconeogenesis form the feeds to PPP and produce xylulose-5-phosphate and erythrose-4-phosphate (E4P) via transketolase (TKL) in the first step. Yu et al. [50] proposed a NOPPP in *M. maripaludis* (Fig. 6). They suggested the presence of this pathway based on the zero activities of oxidative enzymes [glucose-6-phosphate dehydrogenase and 6-phosphogluconate dehydrogenase] and high activities of non-oxidative enzymes [transketolase (MMP1113, MMP1115), transaldolase (MMP1308), ribose-5-phosphate 3-epimerase (MMP1114), and ribulose-5-phosphate isomerase (MMP1189)] [41, 50] . Tumbula et al. [67] supported this observation by ruling out oxidative PPP based on the labelling patterns of riboses after supplementing the medium with [2-^13^C] acetate. They argued that E4P cannot be the precursor for aromatic amino acids (AroAAs), if NOPPP is its only route. Therefore, they conjectured an alternative route (carboxylation of a triose such as dihydroxyacetone phosphate) for E4P. Porat et al. [68] on the other hand showed that E4P is not a precursor for AroAAs in *M. maripaludis*. They proposed two alternative routes for the syntheses of AroAAs based on the presence of dehydroquinate dehydratase. The details of these routes are provided in the Additional file 1. NOPPP is mainly regulated by substrate availability [69, 70]. However, no such regulation has been shown yet in *M. maripaludis*.

## Nitrogen metabolism

*M. maripaludis* can utilize three nitrogen sources: ammonia, alanine, and dinitrogen (free N_2_) with ammonia being the most preferred source for growth [47, 71] .

Ammonia assimilation occurs in *M. maripaludis* via glutamine synthetase (encoded by *gln*A) which makes glutamine from glutamate and ammonia [72]. Glutamine then serves as the precursor for protein synthesis. Cohen-Kupiec et al. [72] observed that *gln*A mutants are unable to grow even in the presence of exogenous glutamine and alanine indicating the essentiality of *gln*A and absence of glutamine transporters.

As discussed before, alanine uptake occurs in *M.maripaludis* via alanine racemase and alanine permease [47]. Moore et al. [47] confirmed that alanine converts to pyruvate and ammonia by alanine dehydrogenase.

**Fig. 7:**
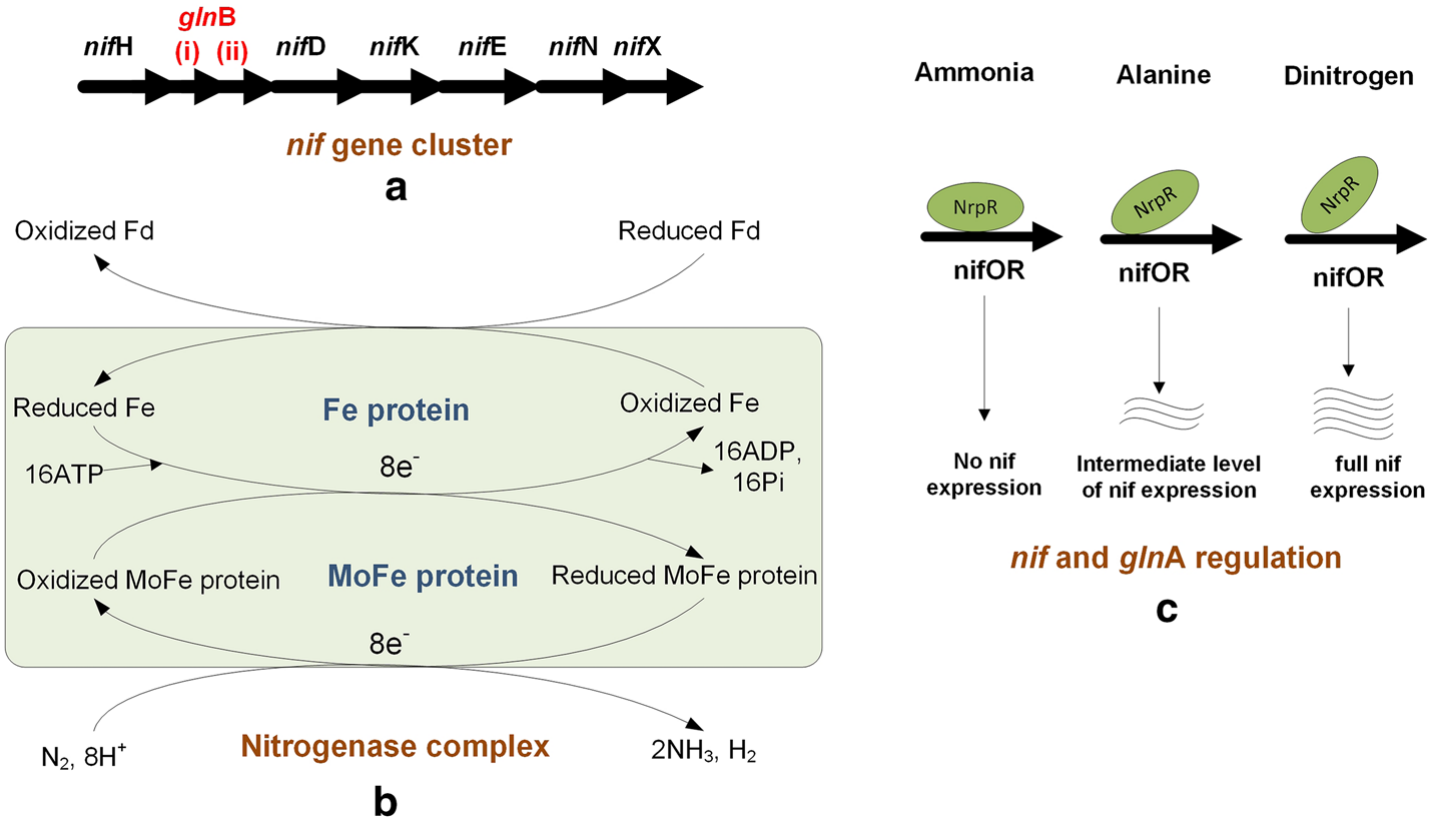
Nitrogen fixation in *M. maripaludis*. **a** The *nif* operon in *M. maripaludis.* **b** Nitrogenase complex. **c** Differential binding of *nif* and *gln*A regulation under different nitrogen substrates/nitrogen limiting conditions. Where, nifOR represents *nif* operators

Free N_2_ fixation or diazotrophy in methanogens including *M. maripaludis* is well established and extensively reviewed in the literature [73–75]. A comparison of four nitrogen-fixing hydrogenotrophic methanococci (*M. formicicus, M.maripaludis, M. aeolicus,* and *M. thermolithotrophicus*) is given in Table 3 [71, 76–78]. Blank et al. [79] showed diazotrophy in wild-type *M. maripaludis* and its four mutants using transposon insertion mutagenesis. Kessler et al. [73] characterized the *nif* gene cluster in *M. maripaludis* based on a sequence analysis. Six *nif* genes (*nif*H, D, K, E, N, and X) and two homologues of bacterial nitrogen sensor-regulator *gln*B (*i & ii*) exist between *nifH* and *nifD* in a single operon in *M. maripaludis* (Fig. 7a). Although the conserved order of *nif* genes resembles that in *Bacteria*, the presence of single operons and two homologues between *nif* genes is unique to *Archaea.*

**Table 3:** A comparison on the characteristics of N_2_-fixing hydrogenotrophs of the order *Methanococcales*

Property	*Methanotorris formicicus*	*Methanococcus maripaludis*	*Methanococcus aeolicus*	*Methanothermococcus thermolithotrophicus*
Type strain	Mc-S-70	JJ	Nankai-3	SN1
Cell diameter (mm)	0.8–1.5	0.9–1.3	1.5–2.0	1.5
Substrates for methanogenesis	H_2_ + CO_2_, formate	H_2_ + CO_2_, formate	H_2_ + CO_2_, formate	H_2_ + CO_2_, formate
Autotrophy	+	+	+	+
Yeast extract stimulates growth	–	+	–	–
Selenium simulates growth	–	+	+	nd
Nitrogen source	NH_3_, N_2_, NO_3_	NH_3_, N_2_, alanine	NH_3_, N_2_	NH_3_, N_2_, NO_3_^−^
Sulfur source	S^2−^	S^2−^, S^0^	S^2−^, S^0^	S^2−^, S^0^, S_2_O_3_^2−^, SO_3_^2−^, SO_4_^2−^
Temperature range (°C)	55–83	<20–45	<20–55	17–70
Temperature optimum (°C)	75	35–40	46	60–65
pH range	6.0–8.5	6.5–8.0	5.5–7.5	4.9–9.8
pH optimum	6.7	6.8–7.2	7	5.1–7.5
NaCl range (%, w/v)	0.4–6.0	0.3–5	0.3–6	0.6–9.4
NaCl optimum (%, w/v)	2.4	0.6–2	1–2	2–4
Genome size (Mb)	1.82	1.66	1.57	1.69
GC content (mol %)	33	33	32	34
Doubling time (hr)	0.5	2	1.3	~1
Source	Deep-sea black smoker chimney	Salt marsh sediments	Marine sediments	Coastal geothermally heated sea sediments
Reference	[78]	[3]	[77]	[130]

Modified from [129]

As shown in Fig. 7b, diazotrophy is effected by multiprotein nitrogenase complex comprising an Fe protein and a MoFe protein [80]. In the presence of N_2_, the oxidized Fe protein reduces by taking electrons from reduced ferredoxins. The reduced Fe protein then oxidizes in the presence of ATP and reduces the MoFe protein. The MoFe protein donates the electrons to N_2_ and reduces it to ammonia in three successive steps: nitrogen to diamine to hydrazine to two ammonia molecules and one H_2_. These reductive steps require electrons from reduced ferredoxins, and their relatively high-energy demand makes N_2_ fixation unfavorable in *M. maripaludis*. Therefore, the cells are less likely to activate this fixation, when ammonia or alanine is available [48, 81].

### Regulation

Cohen-Kupiec et al. [72] hypothesized that both *nif* and *gln*A are regulated by the same nucleotide binding sequences (Fig. 7c) residing in the *nif* promoter region of *M. maripaludis*. Lie et al. [82] confirmed this by isolating NrpR protein. NrpR represses both N_2_ fixation and *gln*A expression by binding to the aforementioned nitrogen operator sequences (nifOR). While NrpR represses *nif* transcription fully in the presence of ammonia, it represses it partially in the presence of alanine, and represses fully in the presence of free N_2_. As discussed before, NrpR in turn is regulated by 2-oxoglutarate levels in the TCA cycle [65]. As shown in Fig. 7c, the binding of NrpR to nifOR weakens during nitrogen deficiency, allowing 2-oxoglutarate to induce *nif* transcription. Experiments have also demonstrated that cell growth with alanine was only marginally lower than with ammonia, while significantly reduced with free nitrogen [48].

*glnB* proteins (i & ii), encoded within the nif gene cluster as shown in Fig. 7a, play a key role in nitrogen sensing and regulation in *M. maripaludis* [81]. *gln*B + strain lost nitrogenase activity within an hour of ammonia addition [83], while *gln*B mutant did not. This indicates that the *glnB* proteins switch off the nitrogenase activity in the presence of ammonia. Kessler et al. [71] reported the importance of molybdenum-dependent nitrogenases during nitrogen fixation in *M. maripaludis*.

The above six subsections along with amino acid and nucleotide metabolisms described in the Additional file 1 complete our picture for the metabolic processes of *M. maripaludis*. In spite of the extensive literature on this organism, we could identify at least a few gaps in our current understanding, which offer opportunities for further research. While a comprehensive picture for the metabolic network and its individual components is useful for the research community, we need to note that all these subsystems reside in a single cell, interact with each other in a complex manner, and result in various cellular phenotypes. Furthermore, the picture so far has been largely qualitative and experimental. As recent research [42, 84–86] has demonstrated the importance of synergizing the experimental with the computational, the qualitative with the quantitative, and the elemental with the systemic, we now complement our picture with a quantitative and systems biology perspective. This is essential for fruitful applications of *M. maripaludis*. However, very little work exists on the systems biology of *M. maripaludis*.

## Molecular biology tools

The 1.6 Mb long *M. maripaludis* genome covers 1722 protein-coding genes with unique hydrogenases [34]. Hendrickson et al. [41] have reported its complete genome sequence and ORF functionalities [87]. Genetic tools are available for manipulating its fully sequenced genome via selectable markers [88], shuttle vectors [89], integrative plasmids and gene replacements [90], and markerless mutagenesis [47].

### Selectable markers

It is difficult to identify antibiotic resistant markers in methanogens due to different ribosome structures and the absence of peptidoglycans in their cell walls. Puromycin resistance in *M. maripaludis* was reported by transforming it with pKAS100 and pKAS102 plasmids [89]. To aid vector transformation, an optimized polyethylene glycol (PEG) method was proposed [91]. This method increased transformation frequency by an order of four to five (2 × 10^5^ transformants/µg of insertion vector) as compared to the natural transformation method. Subsequent methylation of plasmid with PstI methylase increased transformations at least four-folds, thus approaching those obtained for *E. coli*. Addition of divalent cations inhibit transformation [91]. Neomycin is the second selectable marker reported for *M. maripaludis* [88], for which aminoglycoside phosphotransferase genes APH3’I and APH3’II were cloned under the control of *Methanococcus voltae* methyl reductase promoter. 500–1000 µg/ml of neomycin delayed the growth of *M. maripaludis*, and 1000 µg/ml inhibited it completely. Kanamycin and geneticin are non-inhibitory for *M. maripaludis* [92, 93].

### Shuttle vectors

Tumbula et al. [9] constructed a shuttle vector pDLT44 for *M. maripaludis* JJ using plasmid pURB500 (from *M. maripaludis* C5) and pMEB.2 (*E. coli* vector containing a methanococcal puromycin resistance marker). This shuttle vector was found to be stable in *E. coli* under ampicillin selection. This was the first report of a plasmid replicated independently in a methanogen, which can be manipulated in *E. coli*. Although pURB500 was originated from a methanococcus, it did not replicate in *M. voltae*. Another study [94] reported expression shuttle and integrative vectors for *M. maripaludis* using histone promoter (P_*hmvA*_) and multiple cloning sites from *M. voltae* for overexpressing *ilvBN* and *ppsA.* These expression vectors may be useful for studying the physiology and biochemistry of *M. maripaludis.* However, their transformation efficiencies vary from one strain to another [10]. For instance, *M. maripaludis* S2 showed much lower efficiency than *M. maripaludis* JJ, but it can be improved by manipulating a shuttle vector. Walters et al. [10] showed that a significantly smaller shuttle vector pAW42 was sufficient to maintain in *M. maripaludis* S2 and provided 7000-fold increase in transformation efficiency for pURB500-based vectors.

### Integrative plasmid and gene replacement

Stathopoulos et al. [95] constructed an integration vector pIJA03-*cys*S for *M. maripaludis* to determine the essentiality of *cysS* gene coding for cysteinyl-tRNA synthetase. They successfully replaced *cysS* by constructing a pBD1 vector by using another plasmid pPH21310. Several other mutants of *M. maripaludis* have also been constructed using the techniques of integrative plasmids and gene replacement. For example, acetate auxotrophs were isolated by random insertional mutagenesis in the wild type *M. maripaludis* with the help of pWDK104 [96]. Using transposon insertion mutagenesis, mutations were made in and around *nif*H gene to study nitrogen fixing abilities of four transformants. In another study of transposon insertion mutagenesis, an 8-kb region corresponding to the *nif* gene cluster was confirmed for nitrogen fixation [73].

### Markerless mutagenesis

Moore [47] demonstrated markerless mutagenesis in *M. maripaludis* to show the roles of genes with an unusual ability to use d-alanine or l-alanine. They used a negative selection based system with *hpt* and *upt* genes encoding for hypoxanthine and uracil phosphoribosyl transferases present in *M. maripaludis*. The Hpt system was used to produce markerless in-frame deletion mutations in three genes (*ald*, *alr,* and *agcS*) coding for alanine dehydrogenase, alanine racemase, and alanine permease. *hpt* was used together with *upt* to restore the function of wild type *ald*.

## Systems biology

In recent years, systems biology models have been developed for many microbes [97], plants [98], and animals [99] to understand, analyze, and quantify the extent and impact of intracellular interactions and genetic perturbations [100]. However, no such model existed for *M. maripaludis*, until Goyal et al. [101] reported the first constraint-based genome-scale metabolic model (*i*MM518). The model comprised of 570 reactions, 556 metabolites, and 518 genes (30 % ORF coverage) across 52 pathways. The details are available in [101]. The only other model [102] available in the literature is for a coculture of *M. maripaludis* and *D. vulgaris*, developed for studying methane production from lactate, and understanding the syntrophic association between the two. Unlike *i*MM518, this model is limited to the reactions in central metabolism only.

Goyal et al. [101] studied the essentialities of genes and reactions in *M. maripaludis*. Of the 518 genes in their model, 278 proved essential and 240 non-essential. 282 of the 570 reactions proved essential for cell growth. In a previous study, Sarmiento et al. [87] had experimentally analyzed the gene functions in *M. maripaludis* using whole-genome libraries of Tn5 transposon mutant. 34 of the metabolic genes deemed essential for growth by Sarmiento et al. [87] were also in the list of essential genes by Goyal et al. using *i*MM518.

Goyal et al. [101] compared the effectiveness of various carbon, hydrogen, and nitrogen sources on cell growth and methane production. Both their model and our recent experiments (unpublished work) suggest that *M. maripaludis* cannot grow on formate alone (in the absence of CO_2_). *i*MM518 also shows that it cannot grow on acetate alone, but no experiments have proven this. Lupa et al. [23] have reported growth on formate under N_2_/CO_2_ and Wood et al. [103] have reported growth on formate under H_2_/CO_2_. *i*MM518 predicts both. This suggests that both H_2_ and formate can act as electron donors, while CO_2_ is the sole carbon substrate. Between H_2_ and formate, H_2_ is a better hydrogen source (or electron donor) as growth on CO_2_/H_2_ is higher than CO_2_/formate.

Based on *i*MM518, ammonia is better than N_2_ as a nitrogen source for growth, but worse for methane production. Thus, free N_2_ is more advantageous for methane production than ammonia with the added benefit of reduced biomass. The reason seems to be the additional ATPs required by nitrogenases for fixing N_2_ to ammonia first before cellular assimilation. Since methanogenesis is the only energy producing pathway in *M. maripaludis*, the additional energy is supplied by enhanced methanogenesis.

*i*MM518 enabled Goyal et al. [101] to identify the best gene combinations whose deletions would maximize methane production in *M. maripaludis*. Some of their identified targets for single and multiple gene deletions are *adk*A (MMP1031), *acd* (MMP0253), *mdh* (MMP0645), *acd* (MMP0253) & *mdh* (MMP0645), *adk*A (MMP1031) & *mdh* (MMP0645), and *acd* (MMP0253) & *cim*A (MMP1018) & *mdh* (MMP0645).

In addition to the above, *i*MM518 also successfully predicted several experimentally observed phenotypes such as the roles of *leu*A that encodes the first enzyme for leucine biosynthesis, *por*E and/or *por*F whose deletions affect the growth and oxidation of pyruvate, and *nif* and *gln*A expressions during nitrogen availability. With its quantitative power and useful insights into the metabolic processes, such a validated model can help tremendously in reengineering *M. maripaludis* for desired ends.

A recent experimental study [104] measured extracellular fluxes of CO_2_, H_2_, and CH_4_ in *M. maripaludis* during growth on CO_2_ as the sole carbon substrate. Using these fluxes and a systems biology model (in this case, *i*MM518), the study also proposed a procedure for estimating maintenance energy parameters (growth associated maintenance, GAM and non-growth associated maintenance, NGAM).

## Potential applications

Methanogens play a key role in the global carbon cycle by reducing atmospheric CO_2_ [27]. Their unique characteristics in general, and those of *M.maripaludis* in particular, offer potential for applications in wastewater treatment, carbon capture and utilization, value-added chemicals production, GTL (Gas to Liquid) applications, and methane production from renewable hydrogen or via electromethanogenesis. Although *M. maripaludis* has not been used in an industrial setup so far, its attractive features offer much potential for these applications. We now briefly discuss the existing work on each potential application, and assess the promise of *M. maripaludis*.

### Wastewater treatment

Tabatabaei et al. [105] has summarized the characteristics of methanogenic populations used in wastewater treatment. The production of biogas from the anaerobic degradation of waste relies on a symbiotic relationship between syntrophic bacteria (*Syntrophomas*, *Synthrophospora*, and *Syntrophobacter*) and methanogens [106]. The former convert acid-phase products into acetates and H_2_ for use by the latter. New evidence [107–109] also suggests the possibility of direct electron transfer through nanowires/electrically conductive pili between the two. Without the methanogens removing the acetates and H_2_, acetogenesis cannot proceed [110].

Of all the methanococci, *M. maripaludis* has many advantages such as autotrophic growth, short doubling time, N_2_-fixation, and stimulation of growth by acetate/amino acids. Compared to mesophilic methanococci (e.g. *M. vannielli*, *M. voltae*, and *M. aeolicus*), *M. maripaludis* seems a better choice for a pure culture. *M. vannielli* has a long doubling time of 8 h, *M. voltae* does not show autotrophy and requires both acetate and amino acids for its growth, while *M. aeolicus* requires a higher temperature of ~46 °C for optimum growth. The genera such as methanocaldococcus, methanotorris, and methanothermococcus are thermophilic and require even higher temperatures (65–85 °C) for optimum growth. Thus, *M. maripaludis* has an advantage over other methanococci for maintaining a low partial pressure of H_2_ during wastewater treatment at low temperatures.

### Carbon capture and conversion

The flue gas exhausts from power plants typically have 3–15 % CO_2_ in majority N_2_. Global CO_2_ emissions are the major cause for global warming and climate change [111]. While the world’s leading nations have committed to reduce CO_2_ emissions in the future, the possibility of sequestration has been losing favor due to various geological and societal reasons. In such a scenario, the interest in converting CO_2_ to useful products or fuels is increasing. Given the availability of renewable H_2_ from wind, solar, nuclear, etc., *M.maripaludis* with its ability to uptake CO_2_ in the presence of N_2_ offers a potential route to capture and convert CO_2_ simultaneously from CO_2_ emissions to a useful fuel such as methane [83]. *M. maripaludis* in a microbe consortium with other methanogens such as *M. aeolicus* [77]*, M. thermolithotrophicus* [76]*, M. formicicus* [78] can be in principle used as to capture and convert CO_2_ from power and chemical plant emissions. Given that all methanogens are anaerobic, such a possibility does demand some pretreatment of the flue gas emissions that typically contain some residual oxygen. Further studies are clearly needed to test the feasibility and economics of such an application at large scale.

### Methane from renewable energy

The main challenge in using methanogens for large-scale biomethane production is the need for H_2_. The only viable source for this H_2_ is a renewable energy source such as solar, tidal, nuclear, or wind. Formate and H_2_ are being studied [112] as potential options for energy storage to temporally balance the availability of energy with the use of electricity. *M. maripaludis* along with other methanogens offers the potential for converting formate and H_2_ along with CO_2_ into useful fuels such as methane and methanol.

The other possibility is to convert surplus renewable electricity directly into methane via electrochemical methanogenesis. This is already established for a mixed-culture of methanogenic microbes comprising *Methanobacteriumsp.* (>93 %) and *Methanobrevibacter* (~5 %) [113]. Recently, Lohner et al. [22] also demonstrated the uptake of electrons by a hydrogenase-mutant of *M. maripaludis*, although methane production relative to the wild type was only 1/10 as discussed before. However, these studies do point to the potential of methanogens such as *M. maripaludis* as biocatalysts for the electrochemical conversion of CO_2_.

### Hydrogen production

H_2_ production by various aerobic and anaerobic microbes has been reported in the literature [114, 115]. While genetically engineered *E. coli* strains can produce 1.7 µmol/mg DCW min to 4.2 µmol/mg DCW min [116] of H_2_ from formate during growth on glucose, the wild-type *M. maripaludis* S2 can readily produce 1.4 µmol/mg DCW min from formate with only CO_2_ [23]. Thus, scope exists to enhance this production rate further by reengineering *M. maripaludis*.

### Other applications

Several applications remain unexplored for *M. maripaludis* in spite of its unique advantages. For instance, it can be studied for the production of high value-added pharmaceuticals, vitamins, amino acids, corrinoids, and terpenoids. Our recent studies (unpublished work) with *i*MM518 suggests that geraniol, a useful flavoring agent [117], can be produced by up-regulating *hmg*A and/or *idi*1 in *M. maripaludis.* The biotransformation of 2,4,5-trinitrotoluene, a priority pollutant, and metabolic conversion of 5-methylfurfurals and 2-methylfufurals (formed during the concentration of aqueous wastes in the paper and pulp industries) to furfurals have been studied with *Methanococcus spp. (strain B)* [118, 119]. Given the availability of ready genetic tools, such biotransformations can also be explored with *M. maripaludis*. Other possible applications include the production of liquid biofuels such as methanol, butanol, etc., as being explored [120] by some research groups around the world.

## Conclusions

*M. maripaludis* is a model methanogen with some unique metabolic features (e.g. methanogenesis from simple carbon substrates such as CO_2_, glycolysis via modified EMP pathway, conversion of carbon to a fuel rather than accumulation into biomass, whole cell biocatalysis, diazotrophy, synthesis of all 22 amino acids, and RTCA) that other common workhorses such as *E. coli* and yeasts do not. This paper presented an integrated and comprehensive review of its metabolic processes, which is missing in the literature in spite of extensive fundamental research on specific aspects of its biochemistry and genetics. We classified and described the salient features of its eight major subsystems and integrated them into a holistic schematic. Our review suggests that further efforts are required towards understanding regulation, acetate biosynthesis, amino acid synthesis, electron transport chain, glycolysis, value-added chemicals/fuels production, metabolism under stress, carbon capture and utilization applications, and systems biology models. Recent tools for next generation sequencing and -omics, and their integration with systems biology models can deepen our understanding of *M. maripaludis* at the molecular level and promote further research into this interesting microbe. We hope that this review will benefit both modelers and experimentalists to familiarize themselves with *M. maripaludis* as an opportunistic methanogen and pursue further studies on our identified gaps within its biochemical pathways and applications.

## Additional file

10.1186/s12934-016-0500-0 Taxonomy, cell structure, cultivation, amino acid metabolism, nucleotide biosynthesis, and molecular biology tools for *M. maripaludis.*

## Abbreviations

AroAAs: aromatic amino acids
CODH/ACS: carbon monoxide decarbonylase/acetyl-CoA synthase complex
CO_2_: carbon dioxide
CoM: coenzyme M
DCW: dry cell weight
EMP: Embden-Meyerhof-Parnas
E4P: Erythrose-4-phosphate
HCOOH: formate
FBP: fructose-bisphosphatase
GTL: gas to liquid
GK: glucokinase
G3P: glyceraldehyde-3-phosphate
GAPDH: G3P dehydrogenase
GAPOR: G3P ferredoxin oxidoreductase
GAM: growth associated maintenance
H_2_: molecular hydrogen
H_2_O: water
HDR: heterodisulfide reductase
CH_4_: methane
MFR: methanofuran
MTR: methyltransferase
NGAM: non-growth associated maintenance
NOPPP: non-oxidative pentose phosphate pathway
Fd(ox): oxidized ferredoxin
PPS: phosphenolpyruvate synthase
PFK: phosphofructokinase
PEG: polyethylene glycol
PMF: proton-motive force
PYC: pyruvate carboxylase
PORs: pyruvate:ferredoxin oxidoreductases
Fd(rd): reduced ferredoxin
RTCA: reductive tricarboxylic acid cycle
TKL: transketolase
