# Supplementary material for: Metabolic processes of Methanococcus maripaludis and potential applications
Source: Microb Cell Fact. 2016 Jun 10;15:107. doi: 10.1186/s12934-016-0500-0 (PMC4902934; doi:10.1186/s12934-016-0500-0)
Supplement: Supplementary file 1 — 10.1186/s12934-016-0500-0 Taxonomy, cell structure, cultivation, amino acid metabolism, nucleotide biosynthesis, and molecular biology tools for M. maripaludis. [file 12934_2016_500_MOESM1_ESM.docx]

**TAXONOMY AND CELL CULTIVATION**

**Identification and classification**

In late 1970s, 16S rRNA gene sequences were adopted for phylogenetic analyses, and *Archaea* was classified as the third domain of life by Carl Woese and his colleagues [[1](#_ENREF_1)]. *Archaea* is a diverse group of microorganisms widely distributed in extreme habitats, such as hot springs for the phylum Crenarchaeota and salt lakes for the phylum Euryarchaeota. The phylum Euryarchaeota includes eight classes: Methanobacteria, Methanococci, Methanomicrobia, Halobacteria, Thermoplasmata, Thermococci, Archaeoglobi, and Methanopyri. The distinctive features of the Methanococci include rapid growth in both mesophilic and thermophilic temperatures, proteinaceous S-layer as the cell envelope, and a nutritional requirement for selenium. The Methanococci include a single order Methanococcales, which includes methanogens characterized by a coccoid shape and the presence of glycolipids and polar lipids in varying compositions [[2](#_ENREF_2)]. The order Methanococcales is composed of two families: *Methanococcaceae* and *Methanocaldococcaceae*. They differ in 12% 16S rRNA sequences and growth temperatures [[3](#_ENREF_3)]. *Methanococcaceae* are extremely thermophilic or mesophilic, while *Methanocaldococcaceae* are all hyperthermophilic. The family *Methanococcaceae* is further subdivided into two genera, namely *Methanococcus* and *Methanothermococcus* based on their different optimum growth temperatures. The core lipid of *Methanococcus* is mainly archaeol and hydroxyarchaeol, while caldarchaeol is additionally present in *Methanothermococcus* [[4](#_ENREF_4)]. The most abundant polyamine is spermidine in *Methanococcus* and *Methanothermococcus*. The genus *Methanococcus* is subdivided into four species, [*M. aeolicus*](http://en.wikipedia.org/w/index.php?title=Methanococcus_aeolicus&action=edit&redlink=1)*,* [*M. maripaludis*](http://en.wikipedia.org/w/index.php?title=Methanococcus_maripaludis&action=edit&redlink=1)*,* [*M. vannielii*](http://en.wikipedia.org/w/index.php?title=Methanococcus_vannielii&action=edit&redlink=1)*, and* [*M. voltae*](http://en.wikipedia.org/w/index.php?title=Methanococcus_voltae&action=edit&redlink=1)*,* based on 16S rRNA sequences, DNA relatedness, cellular protein patterns, and phenotypic methods [[5](#_ENREF_5)]. The detailed characteristics of each species have been described previously [[3](#_ENREF_3)]. *M. maripaludis* species have five sequenced strains: *M. maripaludis* S2, *M. maripaludis* C5, *M. maripaludis* C6, *M. maripaludis* C7, and *M. maripaludis* X1, which were classified based on their 54-69% DNA relatedness and 99.2% sequence similarity with 16S rRNA sequences [[5](#_ENREF_5)].

**Growth media, culture conditions, and storage**

*M. maripaludis* is a routinely cultured hydrogenotrophic methanogen. It can grow anaerobically on minimal medium (McN), complex medium (McC), and minimal medium plus sodium acetate (McNA) [[6](#_ENREF_6)]. Plate colonization of *M. maripaludis* on solidified agar medium with 50-100% plating efficiency and maximum colony size was achieved by optimizing the inoculation method, H_2_S concentration, and agar moisture content [[7](#_ENREF_7)]. The preferred carbon and energy source is CO_2_-H_2_ (20:80 v/v) at a pressure of about 275 $kPa$. In the absence of H_2_, formate can serve as the sole carbon and energy source in the presence of N_2_-CO_2_ (80:20 v/v) [[8](#_ENREF_8), [9](#_ENREF_9)]. However, excess of either H_2_ or formate decreases the ratio of growth yield to methane significantly [[10](#_ENREF_10)].

Amino acids and vitamins may affect [[11](#_ENREF_11)] the growth rate and cell yield (mg dry cell weight/ml) of methanococci. Some of the facultative autotrophic strains, such as Strain D1, Strain C5, Strain C9, and Strain C14, assimilated large amounts of amino acids, but the growths of *M. vannielli* and *M. aeolicus* were unaffected. The growth of *M. maripaludis* was moderately stimulated, possibly because *M. maripaludis* was capable of utilizing alanine as the sole nitrogen source in the absence of other nitrogen sources, while *M. vannielli* and *M. aeolicus* could not use alanine as the sole nitrogen source. Although exogenous amino acids can substitute for 40-60% of the total cell carbon by some autotrophic methanococci, they were not the major nitrogen sources and were not extensively metabolized. The growth of 16 autotrophic isolates, such as *M. maripaludis*, *M. vannielii*, *M. deltae*, and *M. aeolicus*, remained unaffected by the mixture of water soluble vitamins [[11](#_ENREF_11)]. Pantothenate was the only vitamin that stimulated the growth of *M. voltae* [[12](#_ENREF_12)].

Long-term storage of *M. maripaludis* cultures in glycerol stock solutions has been reported [[6](#_ENREF_6)]. Cultures were grown to the early stationary phase, concentrated by centrifugation, and cell pellets were resuspended in media containing 25% glycerol and then stored at $-$70°C. The addition of glycerol stabilizes frozen microorganisms, prevents damage to cell membranes, and keeps the cell alive for many years.

**AMINO ACID METABOLISM**

*M. maripaludis* is an autotrophic organism that synthesizes all amino acids required for its growth. Sequence comparisons of archaebacterial genes suggest that amino acid biosynthetic enzymes in *Archaea* share a common ancestry with those in *Eubacteria* and *Eukarya* [[13](#_ENREF_13)]. For instance, the enzymes of branched-chain amino acids (BCAAs) characterized in three methanococci (*M. aeolicus, M. maripaludis, M. voltae*) were found to be functionally homologous to eubacterial and eukaryotic enzymes with respect to molecular weight, optimum pH, and kinetic properties [[13](#_ENREF_13)]. Usually, 22 amino acids are required for protein synthesis. 20 amino acids are encoded by the universal genetic codes and the remaining two (selenocysteine and pyrrolysine) are incorporated by unique mechanisms. The 20 amino acids are classified into six groups based on their structures and chemical characteristics of the R group.

In *Methanococcus spp*., the biosynthesis of BCAAs (valine, isoleucine, and leucine) was demonstrated via enzymatic assays [[13](#_ENREF_13)]. Except *M. voltae* that requires leucine, isoleucine, and acetate for its growth, most species grow autotrophically and possess all the genes and enzymes required for growth. Four enzymes (acetohydroxy acid synthase, acetohydroxy acid isomeroreductase, dihydroxy acid dehydratase, and transaminase B) are required for the syntheses of valine and isoleucine, where 2-ketoisovalerate, an intermediate in valine biosynthesis, acts as a precursor for leucine biosynthesis [[13](#_ENREF_13)].

The synthesis of alanine occurs from pyruvate via alanine dehydrogenase [[14](#_ENREF_14)]. Alanine can also serves as the sole nitrogen source during growth [[15](#_ENREF_15)].

Usually, glycine is synthesized from L-serine via serine hydroxymethyltransferases. *M. thermoautotrophicus* showed the presence of serine hydroxymethyltransferases in the genome [[16](#_ENREF_16)], but no homologue for this enzyme is present in *M. maripaludis*. Further studies are required to discover the glycine biosynthesis route in methanococci.

The established route of proline biosynthesis involves glutamic acid to proline conversion, but three enzymes of this pathway are absent in most *Archaea* [[17](#_ENREF_17)]. Graupner *et al.* [[18](#_ENREF_18)] demonstrated the synthesis of proline in *M. jannaschii* from cyclization of ornithine via ornithine cyclodeaminase, while no such enzyme has been characterized in *M. maripaludis*, which suggests a possibility of alternate routes.

The biosynthesis of AroAAs (phenylalanine, tyrosine, and tryptophan) is well understood in methanococci. Chorismate acts as the branch point for phenylalanine, tyrosine, and tryptophan synthesis. Unlike *Bacteria*, E4P is not a precursor for chorismate in *M. maripaludis* and an alternate route has been proposed based on the presence of dehydroquinate dehydratase [[19](#_ENREF_19)]. In this pathway, chorismate is synthesized from 3-dehydroquinate (DHQ) via shikimate pathway. For DHQ synthesis, *M. maripaludis* uses 6-deoxy-5-ketofructose 1-phosphate (DKFP), synthesized after the condensation of methylglyoxal and fructose-1,6-bisphosphate, and L-aspartate-semialdehyde to form 2-amino-3,7-dideoxy-D-threo-hept-6-ulosonate (ADTH), which cyclizes to DHQ [[20](#_ENREF_20)]. DHQ is not only the precursor for chorismate synthesis, but also acts as a precursor for p-aminobenzoic acid (PABA) synthesis. PABA is an intermediate in *M. maripaludis* during the synthesis of tetrahydromethanopterin, one of the cofactors. [[20](#_ENREF_20)]. Two biosynthetic routes for the production of AroAAs from chorismate in *M. maripaludis* were proposed previously [[21](#_ENREF_21)]. To confirm the presence of *de novo* pathway, deletion strains of MMP1394 encoding 3-dehydroquinate dehydratase were constructed and the mutants were auxotrophic for all three AroAAs and no DHQ activity was detected. To evaluate aryl acid dependent pathway, acids were supplemented to the medium to fulfill the requirements of AroAAs in *M. maripaludis*.

Whole genome sequencing studies on *M. maripaludis* provide information on enzymes / ORFs participating in amino acid biosynthesis. The results indicate that all genes required for the biosynthesis of histidine (*his*A, B, C, D, E, F, G, H, I) were present in *M. maripaludis* except *his*J that encodes for histidinol phosphate phosphatase [[18](#_ENREF_18)]. Fondi *et al.* [[22](#_ENREF_22)] further evaluated the biosynthesis of histidine in *Archaea* and suggested that *his* operon might have been assembled multiple times during evolution because *his* genes scattered throughout the genome. In addition, they showed the existence of *his*N gene for histidinol phosphate phosphatase and *his*B gene for imidazoleglycerol-phosphate dehydratase catalyzing sixth and eight steps of the histidine biosynthesis, which suggests that different molecular mechanisms in *M. maripaludis* may drive piece-wise operon formation for histidine biosynthesis.

Lysine biosynthesis in *M. maripaludis* occurs via diaminopimelate aminotransferase (DapL) pathway [[23](#_ENREF_23)]. A mutant of *dap*L homolog in *M. maripaludis* resulted in lysine auxotrophy and suggested that *dap*L is essential for lysine biosynthesis.

The specific activities of arginine biosynthetic enzymes in methanogenic *Archaea* was reported by Meile *et al.* [[24](#_ENREF_24)]. They also mentioned that although biosynthesis sequences were similar in all microbes, differences existed in reaction steps and regulations of this pathway. Arginine biosynthesis and associated genes / enzymes are well characterized in *M. maripaludis* [[18](#_ENREF_18)]*.* The functional conservation of argininosuccinate lyase encoded by *arg*H (catalyze final step in arginine biosynthesis pathway) between *M. maripaludis* and the corresponding *Bacteria* and *Eukarya* was demonstrated [[25](#_ENREF_25)].

Glutamate is synthesized in *M. maripaludis* via glutamate synthase [[26](#_ENREF_26)]. Aspartate is synthesized by aspartate aminotransferase and asparagine glutamine-hydrolyzing asparagine synthase (*asnB*). ^14^C labeling studies on *M. barkeri* and other *Archaea* showed that synthesis of alanine, aspartate, and glutamate occurred from pyruvate, oxaloacetate, and α-ketoglutarate respectively [[27](#_ENREF_27)].

^13^C labeling of serine was consistent with its synthesis from pyruvate via 3- phosphoglycerate. *M. maripaludis* contained *serA* and *serB*, but not a homolog for *serC* [[18](#_ENREF_18)]. Similarly, a homolog for glycine hydroxymethyltransferase encoded by *glyA* is absent for synthesis of glycine from serine.

Stathopoulos *et al.* [[28](#_ENREF_28)] challenged the notion that all 20 aminoacyl-tRNA synthetases are essential for the viability of a cell. They knocked out *cysS* gene encoding cysteinyl-tRNA synthetase (CysRS) from *M. maripaludis* and showed that pure M. maripaludis prolyl-tRNA synthetase (ProRS) can form cysteinyl-tRNA (Cys-tRNA), implying dual-specificity of enzyme for the loss of CysRS. The report by Stathopoulos *et al.* [[28](#_ENREF_28)] was incorrect. While ProRS has low levels of CysRS activity, it is not physiologically significant. Sauerwald *et al.* [[29](#_ENREF_29)] demonstrated this by observing that CysRS mutants could not incorporate exogenous cysteine into cellular protein. The tRNA-dependent cysteine biosynthesis pathway in *M. maripaludis* is well established and occurs in two steps: First, O-phosphoseryl-tRNA ligase (SepRS) aminoacylates uncharged Cys-tRNA with 3-phospho-L-serine (Sep) to form O-phosphoseryl-tRNA (Sep-tRNA). Second, Sep-tRNA:Cys-tRNA synthase (SepCysS) transforms Sep to cysteine [[29](#_ENREF_29)]. Zhang *et al.* [[30](#_ENREF_30)] showed that these two enzymes (SepRS and SepCysS) form a stable binary complex and promote the conversion of intermediate Sep-tRNA to cysteinyl-tRNA by sequestering the binding of the intermediate to elongation factor EF-1α or infiltrating into the ribosome.

Methionine biosynthesis in *M. maripaludis* is unclear. Only one ORF, MMP0401, has been identified in *M. maripaludis* via sequencing studies [[18](#_ENREF_18)] which indicates the synthesis of methionine from homocysteine. The presence of cystathionine β-lyase (*metC*) in *M. maripaludis* indicates the possibility that *M. maripaludis* synthesizes homocysteine (the intermediate precursor of methionine) either by transsulfuration route with cystathione or via direct sulfhydrylation of O-acetylhomoserine [[23](#_ENREF_23)]. Such gaps in the literature need further investigation of existing routes or novel biosynthetic routes.

Selenocysteine is the selenium containing 21^st^ amino acid that is co-translationally incorporated into proteins, which are known as selenoproteins. The amino acid is coded by UGA, which is normally a termination codon during protein synthesis. Only 20% of *Bacteria* and 10% of *Archaea* (Methanococcus, Methanocaldococcus, and Methanopyrus spp.) were found to have machinery for Sec insertion. In *Eukarya*, sec insertion machinery is common in lower organisms, such as green algae and moulds [[31](#_ENREF_31)]. Yuan *et al.* proposed tRNA^Sec^-dependent conversion of *O*-phosphoserine (Sep) to selenocysteine in *Eukarya* and *Archaea* [[32](#_ENREF_32)]. Genetic analysis of selenocysteine biosynthesis in *M. maripaludis* is reported in the literature [[33-35](#_ENREF_33)]

Pyrrolysine is the 22^nd^ natural amino acid and is a lysine derivative encoded by UAG. It is used by methanogenic *Archaea* and was discovered in 2002 at the active site of methylamine methyltransferase in *M. barkeri* [[36](#_ENREF_36)]. *pyl*T gene, whose tRNA product has CUA anticodon, translates to UAG codon as pyrrolysine in some methanogens. The presence of pyrrolysine in *M. maripaludis* is not established, although it has been mentioned in non-pyl-utilizing *Archaea* in the literature [[37](#_ENREF_37)].

**NUCLEOTIDE METABOLISM**

Nucleotides include purines (adenine and guanine) and pyrimidines (thymine and cytosine). In RNA, adenine base pairs with uracil instead of thymine. The purine and pyrimidine biosynthesis in *M. maripaludis* is very well understood and sequencing study showed the presence of related genes involved in biosynthesis of purines and pyrimidines [[18](#_ENREF_18)]. 5-phospho-α-D-ribose 1-diphosphate (PRPP) is synthesized from ribose-5-phosphate with the help of PRPP synthetase and combines with glutamine to form 5-phosphoribosylamine, which goes through a series of reactions and forms inosinic acid (IMP). IMP acts as the branch point for adenosine monophosphate (AMP) and guanosine monophosphate (GMP) biosynthesis in the pathway. AMP is synthesized from IMP and adenylosuccinate and converted to other purine nucleotides, such as adenosine diphosphate (ADP) and adenosine triphosphate (ATP). Similarly, xanthosine 5'-monophosphate (XMP) is synthesized from IMP with NAD^+^ or NADP^+^ as acceptors, which is subsequently converted to other purine nucleotides, such as GMP, guanosine diphosphate (GDP), and guanosine triphosphate (GTP). Pyrimidine biosynthesis converts bicarbonate, L- glutamine, ororate, and PRPP, to uridine monophosphate (UMP) for further conversion to uridine triphosphate (UTP) and cytidine triphosphate (CTP) for participation in nucleic acid biosynthesis.

**References**

1. Liu Y, Whitman WB: **Metabolic, phylogenetic, and ecological diversity of the methanogenic archaea.** *Ann N Y Acad Sci* 2008, **1125:**171-189.

2. Liu Y: **Methanococcales.** In *Handbook of Hydrocarbon and Lipid Microbiology.* Springer; 2010: 573-581.

3. Whitman W, Jeanthon C: **Methanococcales.** In *The Prokaryotes.* Springer New York; 2006: 257-273.

4. Koga Y, Morii H, Akagawa-Matsushita M, OHGA M: **Correlation of polar lipid composition with 16S rRNA phylogeny in methanogens. Further analysis of lipid component parts.** *Bioscience, biotechnology, and biochemistry* 1998, **62:**230-236.

5. Keswani J, Orkand S, Premachandran U, Mandelco L, Franklin M, Whitman W: **Phylogeny and taxonomy of mesophilic *Methanococcus spp.* and comparison of rRNA, DNA hybridization, and phenotypic methods.** *International journal of systematic bacteriology* 1996, **46:**727-735.

6. Whitman WB, Shieh J, Sohn S, Caras DS, Premachandran U: **Isolation and characterization of 22 mesophilic methanococci.** *Systematic and applied microbiology* 1986, **7:**235-240.

7. Apolinario EA, Sowers KR: **Plate colonization of *Methanococcus maripaludis* and *Methanosarcina thermophila* in a modified canning jar.** *FEMS microbiology letters* 1996, **145:**131-137.

8. Costa KC, Lie TJ, Jacobs MA, Leigh JA: **H2-independent growth of the hydrogenotrophic methanogen Methanococcus maripaludis.** *MBio* 2013, **4**.

9. Lupa B, Hendrickson EL, Leigh JA, Whitman WB: **Formate-dependent H_2_ production by the mesophilic methanogen *Methanococcus maripaludis*.** *Appl Environ Microbiol* 2008, **74:**6584-6590.

10. Costa KC, Yoon SH, Pan M, Burn JA, Baliga NS, Leigh JA: **Effects of H2 and formate on growth yield and regulation of methanogenesis in Methanococcus maripaludis.** *J Bacteriol* 2013, **195:**1456-1462.

11. Whitman WB, Sohn S, Kuk S, Xing R: **Role of amino acids and vitamins in nutrition of mesophilic *Methanococcus spp*.** *Applied and environmental microbiology* 1987, **53:**2373-2378.

12. Whitman W, Ankwanda E, Wolfe R: **Nutrition and carbon metabolism of Methanococcus voltae.** *Journal of bacteriology* 1982, **149:**852-863.

13. Xing R, Whitman WB: **Characterization of enzymes of the branched-chain amino acid biosynthetic pathway in *Methanococcus spp*.** *Journal of bacteriology* 1991, **173:**2086-2092.

14. Moore BC, Leigh JA: **Markerless mutagenesis in Methanococcus maripaludis demonstrates roles for alanine dehydrogenase, alanine racemase, and alanine permease.** *J Bacteriol* 2005, **187:**972-979.

15. Lie TJ, Leigh JA: **Regulatory Response of Methanococcus maripaludis to Alanine, an Intermediate Nitrogen Source.** *Journal of Bacteriology* 2002, **184:**5301-5306.

16. Smith DR, Doucette-Stamm LA, Deloughery C, Lee H, Dubois J, Aldredge T, Bashirzadeh R, Blakely D, Cook R, Gilbert K: **Complete genome sequence of *Methanobacterium thermoautotrophicum* deltaH: functional analysis and comparative genomics.** *Journal of Bacteriology* 1997, **179:**7135.

17. Graupner M, White RH: ***Methanococcus jannaschii* Generates L-Proline by Cyclization of L-Ornithine.** *Journal of bacteriology* 2001, **183:**5203-5205.

18. Hendrickson EL, Kaul R, Zhou Y, Bovee D, Chapman P, Chung J, Conway de Macario E, Dodsworth JA, Gillett W, Graham DE, et al: **Complete genome sequence of the genetically tractable hydrogenotrophic methanogen Methanococcus maripaludis.** *J Bacteriol* 2004, **186:**6956-6969.

19. Porat I, Waters BW, Teng Q, Whitman WB: **Two biosynthetic pathways for aromatic amino acids in the archaeon Methanococcus maripaludis.** *Journal of bacteriology* 2004, **186:**4940-4950.

20. Porat I, Sieprawska-Lupa M, Teng Q, Bohanon FJ, White RH, Whitman WB: **Biochemical and genetic characterization of an early step in a novel pathway for the biosynthesis of aromatic amino acids and p-aminobenzoic acid in the archaeon *Methanococcus maripaludis*.** *Mol Microbiol* 2006, **62:**1117-1131.

21. Porat I, Waters BW, Teng Q, Whitman WB: **Two biosynthetic pathways for aromatic amino acids in the archaeon *Methanococcus maripaludis*.** *J Bacteriol* 2004, **186:**4940-4950.

22. Fondi M, Emiliani G, Lio P, Gribaldo S, Fani R: **The evolution of histidine biosynthesis in archaea: insights into the his genes structure and organization in LUCA.** *J Mol Evol* 2009, **69:**512-526.

23. Liu Y: **Adaptations of Methanococcus maripaludis to its unique lifestyle.** The University of Georgia2010.

24. Meile L, Leisinger T: **Enzymes of arginine biosynthesis in methanogenic bacteria.** *Experientia* 1984, **40:**899-900.

25. Cohen‐Kupiec R, Kupiec M, Sandbeck K, Leigh J: **Functional conservation between the argininosuccinate lyase of the archaeon *Methanococcus maripaludis* and the corresponding bacterial and eukaryal genes.** *FEMS microbiology letters* 1999, **173:**231-238.

26. Cohen‐Kupiec R, Marx Ca, Leigh J: **Function and regulation of glnA in the methanogenic archaeon *Methanococcus maripaludis.*** *J Bacteriol* 1999, **18:**256-261.

27. Kenealy WR, Zeikus J: **One-carbon metabolism in methanogens: evidence for synthesis of a two-carbon cellular intermediate and unification of catabolism and anabolism in *Methanosarcina barkeri*.** *Journal of bacteriology* 1982, **151:**932-941.

28. Stathopoulos C, Kim W, Li T, Anderson I, Deutsch B, Palioura S, Whitman W, Soll D: **Cysteinyl-tRNA synthetase is not essential for viability of the archaeon *Methanococcus maripaludis*.** *Proc Natl Acad Sci U S A* 2001, **98:**14292-14297.

29. Sauerwald A, Zhu W, Major TA, Roy H, Palioura S, Jahn D, Whitman WB, Yates 3rd JR, Ibba M, Söll D: **RNA-dependent cysteine biosynthesis in archaea.** *Science* 2005, **307:**1969-1972.

30. Zhang C-M, Liu C, Slater S, Hou Y-M: **Aminoacylation of tRNA with phosphoserine for synthesis of cysteinyl-tRNA^Cys^.** *Nature structural & molecular biology* 2008, **15:**507-514.

31. Turanov AA, Xu X-M, Carlson BA, Yoo M-H, Gladyshev VN, Hatfield DL: **Biosynthesis of selenocysteine, the 21^st^ amino acid in the genetic code, and a novel pathway for cysteine biosynthesis.** *Advances in Nutrition: An International Review Journal* 2011, **2:**122-128.

32. Yuan J, Palioura S, Salazar JC, Su D, O'Donoghue P, Hohn MJ, Cardoso AM, Whitman WB, Söll D: **RNA-dependent conversion of phosphoserine forms selenocysteine in eukaryotes and archaea.** *Proceedings of the National Academy of Sciences* 2006, **103:**18923-18927.

33. Hohn MJ, Palioura S, Su D, Yuan J, Söll D: **Genetic analysis of selenocysteine biosynthesis in the archaeon *Methanococcus maripaludis*.** *Molecular microbiology* 2011, **81:**249-258.

34. Stock T, Selzer M, Connery S, Seyhan D, Resch A, Rother M: **Disruption and complementation of the selenocysteine biosynthesis pathway reveals a hierarchy of selenoprotein gene expression in the archaeon *Methanococcus maripaludis*.** *Mol Microbiol* 2011, **82:**734-747.

35. Rother M, Mathes I, Lottspeich F, Böck A: **Inactivation of the selB gene in *Methanococcus maripaludis*: effect on synthesis of selenoproteins and their sulfur-containing homologs.** *Journal of bacteriology* 2003, **185:**107-114.

36. Srinivasan G, James CM, Krzycki JA: **Pyrrolysine encoded by UAG in Archaea: charging of a UAG-decoding specialized tRNA.** *Science* 2002, **296:**1459-1462.

37. Alkalaeva E, Eliseev B, Ambrogelly A, Vlasov P, Kondrashov FA, Gundllapalli S, Frolova L, Söll D, Kisselev L: **Translation termination in pyrrolysine-utilizing archaea.** *FEBS letters* 2009, **583:**3455-3460.
